# Immunomodulatory effects and mechanisms of the extracts and secondary compounds of *Zingiber* and *Alpinia* species: a review

**DOI:** 10.3389/fphar.2023.1222195

**Published:** 2023-07-18

**Authors:** Yuandani, Ibrahim Jantan, Md. Areeful Haque, Ade Sri Rohani, Sony Eka Nugraha, Emil Salim, Abdi Wira Septama, Nur Aira Juwita, Nur Aini Khairunnisa, Halimah Raina Nasution, Dinda Sari Utami, Sarah Ibrahim

**Affiliations:** ^1^ Faculty of Pharmacy, Universitas Sumatera Utara, Medan, Indonesia; ^2^ Centre of Excellence for Chitosan and Advanced Materials, Universitas Sumatera Utara, Medan, Indonesia; ^3^ Institute of Systems Biology, Universiti Kebangsaan Malaysia, Bangi, Selangor, Malaysia; ^4^ Department of Symptom Research, The University of Texas MD Anderson Cancer Center, Houston, TX, United States; ^5^ Research Center for Pharmaceutical Ingredients and Traditional Medicine, National Research and Innovation Agency (BRIN), Kawasan PUSPIPTEK Serpong, Tangerang Selatan, Bogor, Indonesia

**Keywords:** *Zingiber* species, *Alpinia* species, gingerols, zerumbone, immunomodulatory, signaling pathways

## Abstract

*Zingiber* and *Alpinia* species (family: Zingiberaceae) are popularly used in food as spices and flavoring agents and in ethnomedicine to heal numerous diseases, including immune-related disorders. However, their ethnomedicinal uses have not been sufficiently supported by scientific investigations. Numerous studies on the modulating effects of plants and their bioactive compounds on the different steps of the immune system have been documented. This review aimed to highlight up-to-date research findings and critically analyze the modulatory effects and mechanisms of the extracts and secondary compounds of several *Zingiber* and *Alpinia* species, namely, *Zingiber officinale* Roscoe, *Z. cassumunar* Roxb., *Z. zerumbet* (L.) Roscoe ex Sm., *Alpinia galanga* Linn., *A. conchigera* Griff, *A. katsumadai* Hayata, *A. oxyphylla* Miq., *A. officinarum* Hance, *A. zerumbet* (Pers.) Burtt. et Smith, and *A. purpurata* (Viell.) K. Schum. on the immune system, particularly via the inflammation-related signaling pathways. The immunomodulating activities of the crude extracts of the plants have been reported, but the constituents contributing to the activities have mostly not been identified. Among the extracts, *Z. officinale* extracts were the most investigated for their *in vitro*, *in vivo*, and clinical effects on the immune system. Among the bioactive metabolites, 6-, 8-, and 10-gingerols, 6-shogaol, and zerumbone from *Zingiber* species and cardamomin, 1′-acetoxychavicol acetate, yakuchinone, rutin, 1,8-cineole, and lectin from *Alpinia* species have demonstrated strong immunomodulating effects. More experimental studies using cell and animal models of immune-related disorders are necessary to further understand the underlying mechanisms, together with elaborate preclinical pharmacokinetics, pharmacodynamics, bioavailability, and toxicity studies. Many of these extracts and secondary metabolites are potential candidates for clinical development in immunomodulating agents or functional foods to prevent and treat chronic inflammatory disorders.

## 1 Introduction

The immune response against pathogens and non-pathogens is regulated by key signaling pathways. Appropriate control of the immune response is required to prevent either hyperresponsive or inadequate responses, which are harmful to the host body that may lead to immune-related diseases (Chaplin, 2010). The innate immune response involves several signaling pathways, including the toll-like receptor (TLR) and the stimulator of interferon genes (STING) pathways. Upon activation, TLRs recruit toll/interleukin-1 receptor (TIR)-domain-containing adaptor proteins, which provide receptor sites for relevant proteins and initiate numerous signaling processes by facilitating the phosphorylation of IkB-α to activate nuclear factor-kappa β (NF-κB), leading to a variety of inflammatory cytokines transcription. STING is an intracellular signaling protein that, upon activation, stimulates type I interferon (IFN) production and other inflammatory mediators (Du et al., 2016; Haag et al., 2018). Specific immunity response is also initiated by binding of antigen to receptors of B and T lymphocytes by major histocompatibility complex (MHC)-mediated antigen presentation, which stimulates multiple signaling cascades in both B and T cells. This interaction stimulates the activation of helper T cells. However, cytotoxic T cells are activated if the antigen-presenting cell (APC), such as the dendritic cell, is infected with a virus and viral proteins are produced and displayed on the surface along with class I MHC proteins (Chapel et al., 2005). Antigen binding by the B-cell receptor (BCR) activates several signaling cascades, including GTPases, transcription factors, and kinases, resulting in a change in cell metabolism, cytoskeletal structure, and gene expression. BCR signaling complexity enables several outcomes, including survival, proliferation, apoptosis, and differentiation into memory B cells or plasma cells (Harwood and Batista, 2009).

**FIGURE 1:**
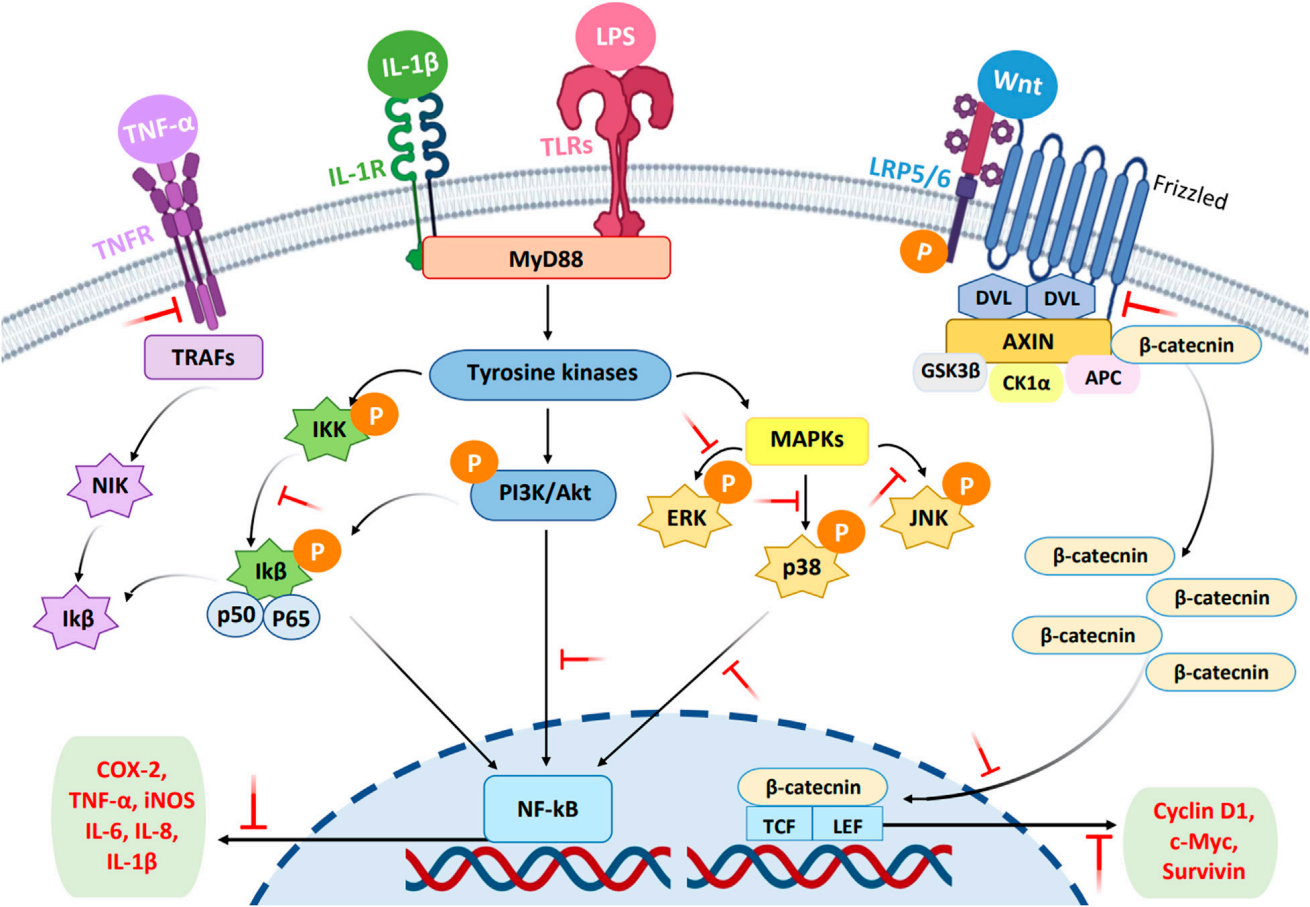
Possible inhibitory effects of phytochemicals on the cell signaling pathways (MAPKs, PI3K/Akt, NF-κB, and Wnt). The blocking of target proteins in the signal pathways by the compounds is represented by short T-shaped red lines. NF-κB, nuclear factor-kappa β; PI3K/Akt, phosphatidylinositol 3-kinase and protein kinase B; MAPK, mitogen-activated protein kinase; Wnt, Wnt/β-catenin; P, phosphoryl group.

Cell signaling networks play a necessary function in the etiology of numerous diseases by stimulating cell survival, proliferation, and apoptosis (Corrales et al., 2015). Targeting cell signaling pathways has recently been regarded as a promising and attractive strategy for discovering new drug leads. The modulating effects of compounds on the immune response via signaling networks cause different expression levels of cytokines, chemokines, acute phase proteins, anti-apoptotic proteins, cell-adhesion molecules, and other inflammatory mediators (de Souza et al., 2012). In various cellular events, mechanisms for intracellular cell signaling play an important and specific role in chronic inflammatory diseases. Inhibition of these pathways is a possible target to provide a better alternative compared to current treatment strategies. Currently, several chemical immunomodulators are used to treat various inflammatory disorders. However, safer and more effective drugs are needed to replace commercial drugs because most of them have side effects. For example, the chronic use of non-steroidal anti-inflammatory drugs (NSAIDs) may lead to gastric mucosal damage, whereas there are various side effects of the immunosuppressive drugs and corticosteroids, such as increased skin fragility and reduced bone marrow (Jantan et al., 2021). Several plants have demonstrated the ability to regulate immune signaling networks. Polyphenols are found to have modulatory effects on mitogen-activated protein kinases (MAPKs), NF-κB, phosphatidylinositol 3-kinase (PI3K/Akt), and Wnt/β-catenin networks and prevent the occurrence of inflammatory disorders (Jantan et al., 2021). Figure 1 depicts the possible modulatory effects of plant secondary metabolites on the cell signaling pathways. The bioactive compounds modulate cell activity through selective actions on the intracellular signaling pathways of various components, particularly cytokines and proinflammatory proteins, which play important cellular functions (Miguel et al., 2014).

Zingiberaceae, the biggest family in the order Zingiberales, consists of approximately 53 genera and over 1,300 species, including *Zingiber*, *Alpinia*, *Etlingera*, *Curcuma*, *Globba*, *Renealmia*, *Riedelia*, *Amomum*, *Aframomum*, *Boesenbergia*, *Hedychium*, *Hornstedia*, and *Meisteria* (Kress et al., 2005; Britannica, 2020). The family is abundantly spread in most subtropical and tropical regions of the globe. Members of this family are typically perennials with sympodial (forked) fleshy rhizomes (underground stems) (Ewon and Bhagya, 2019). The majority of the Zingiberaceae species, including *Zingiber* and *Alpinia* species, such as *Z. officinale* Roscoe, *Z. zerumbet* (L.) Roscoe ex Sm., *Z. cassumunar* Roxb., *Alpinia galanga* Linn., *A. conchigera* Griff, *A. katsumadai* Hayata, *A. oxyphylla* Miq., *A. zerumbet* (Pers.) Burtt. et Smith, *A. purpurata* (Viell.) K. Schum., and *A. officinarum* Hance have been used in traditional medicine to heal various health problems. A few reviews on the phytochemistry, biology, toxicology, and pharmacology of the *Zingiber* and *Alpinia* genus have been published recently (Arora and Ansari, 2014; Zhang et al., 2016; Sharifi-Rad et al., 2017; Dash et al., 2020; Paramita et al., 2021; Deng et al., 2022; Bitari et al., 2023a; Bitari et al., 2023b; Garza-Cadena et al., 2023). There are a few reviews on the immunomodulatory effects of *Zingiber* species, but a comprehensive update and critical analysis of the studies related to these effects are needed (Harun and Mohamad., 2022; Anurag et al., 2022; Yücel et al., 2022; Ni et al., 2022; Thapa et al., 2021). Recently, we reported on the immunomodulatory effects and mechanisms of *Curcuma* species and their bioactive compounds (Yuandani et al., 2021). This review aimed to highlight up-to-date research findings and critically analyze the ability of *Z. zerumbet*, *Z. officinale*, *Z. cassumunar*, *Alpinia galanga*, *A. conchigera*, *A. katsumadai*, *A. oxyphylla*, *A. zerumbet*, *A. purpurata*, and *A. officinarum* extracts and their secondary metabolites to regulate the immune system mainly via the cell signaling pathways.

## 2 Methods

Updated scientific information on the immunomodulating activities of *Zingiber* species, specifically *Z. zerumbet*, *Z. officinale*, *Z. cassumunar*, and *Alpinia species*, including *A. galanga*, *A. conchigera*, *A. katsumadai*, *A. oxyphylla*, *A. zerumbet*, *A. purpurata*, and *A. officinarum* and their bioactive secondary metabolites, were gathered from 2000 until now. The keywords “Zingiberaceae AND signaling pathways immune system,” some species of Zingiber genus, such as “*Zingiber zerumbet* AND signaling in immune system,” “*Zingiber officinale* AND signaling in immune system,” “*Zingiber cassumunar* AND signaling in immune system,” each species of *Alpinia* genus, such as “*Alpinia galanga* AND signaling in immune system,” “*Alpinia conchigera* AND signaling in immune system,” “*Alpinia katsumadai* AND signaling in immune system,” “*Alpinia oxyphylla* AND signaling in immune system,” “*Alpinia zerumbet* AND signaling in immune system,” “*Alpinia purpurata* AND signaling in immune system,” and “*Alpinia officinarum* AND signaling in immune system” were used. In this review, only published scientific data were used, and references without English-language titles were excluded. A thorough literature search was conducted using published scientific papers from databases such as ACS Publications Today, Frontiers, ScienceDirect, Scopus, Google Scholar, and Wiley Online Library. After a thorough analysis of the information acquired on the immunomodulatory properties of the *Zingiber* and *Alpinia* species, future research directions and pertinent prospects for the bioactive metabolites as potential candidates for the development of new natural immunomodulating agents were highlighted.

## 3 Distribution, taxonomy, and ethnopharmacological uses

**FIGURE 2:**
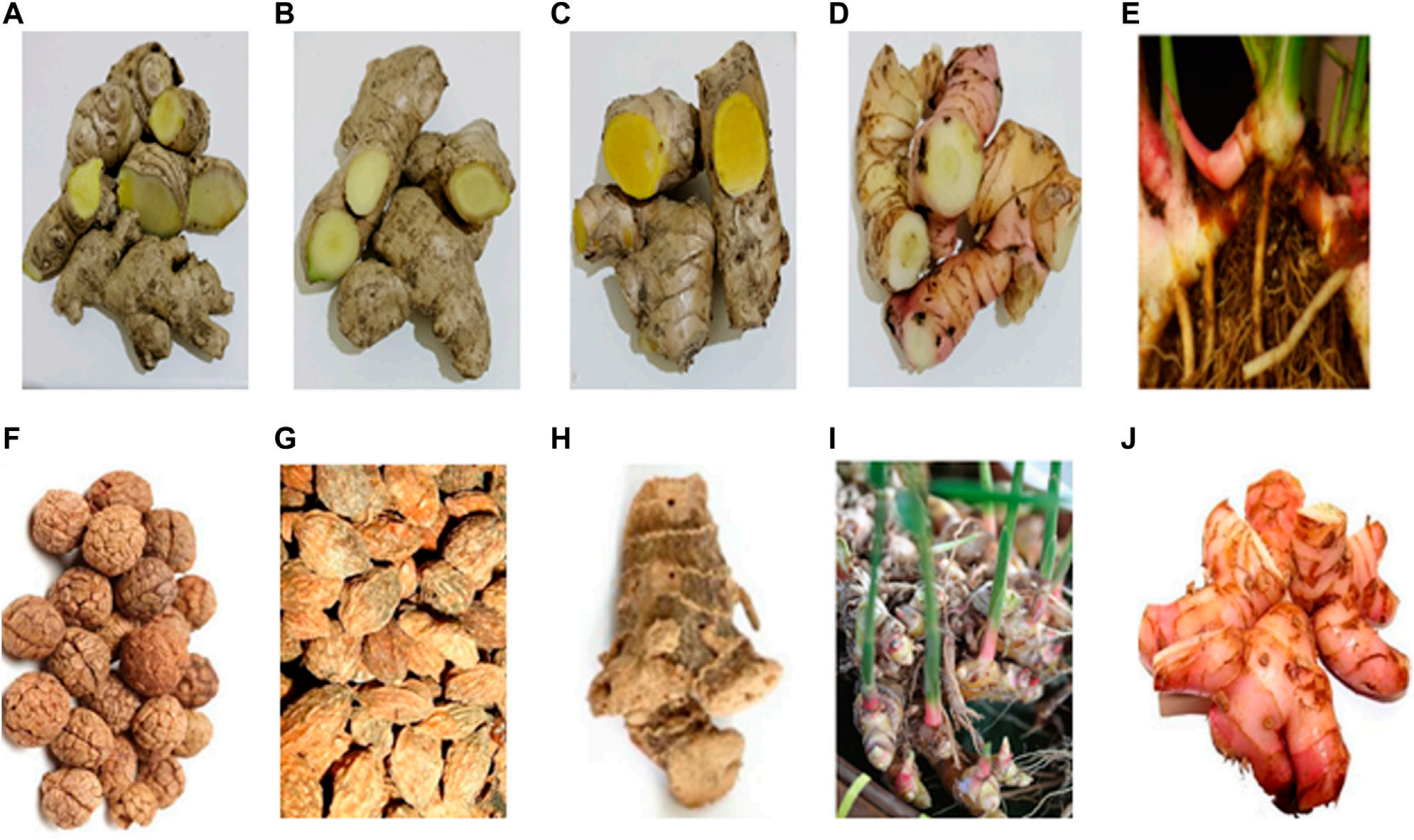
Rhizomes, seed, or kernels: **(A)** *Z. zerumbet*, **(B)** *Z. officinale*, **(C)** *Z. cassumunar*, **(D)** *Z. cassumunar*, **(E)** *A. conchigera*, **(F)** *A. katsumadai*, **(G)** *A. oxyphylla*, **(H)** *A. officinarum*, **(I)** *A. zerumbet*, and **(J)** *A. purpurata*.

*Zingiber* (Zingiberaceae family) is the third largest genera, and more than 141 species of *Zingiber* have been identified. Most genera are aromatic, perennial herbs that grow well in tropical and moist conditions. They are mainly found in tropical and subtropical Asia, Africa, and South America and are commercially cultivated in several countries, including India, China, Indonesia, Thailand, Nigeria, and Philippines (Sharifi-Rad et al., 2017; Deng et al., 2022). The largest genus of the Zingiberaceae family is *Alpinia*, with approximately 230 species. They possess complex taxonomical diversity and are widely distributed in tropical and subtropical areas, including India, China, Indonesia, Australia, Indochina, Indonesia, Japan, Philippines, Sri Lanka, Thailand, and Malaysia (Van et al., 2021). Figure 2 shows the *Zingiber* and *Alpinia* species discussed in this review: *Z. zerumbet*, *Z. officinale*, *Z. cassumunar*, *A. galanga*, *A. conchigera*, *A. katsumadai*, *A. oxyphylla*, *A. officinarum*, *A. zerumbet*, and *A. purpurata.*

*Zingiber officinale* Roscoe is an herbaceous perennial tropical plant that is also extremely adapted to subtropical environments. It thrives in warm and humid environments. The plant is cultivated in many countries, including India, China, Nepal, the United States, Bangladesh, Jamaica, Nigeria, and Taiwan, whereby India is the world’s largest producer (Kumar et al., 2011). It is commonly known as ginger and can be differentiated into three different varieties based on the color and size of the rhizome: big white ginger (*Z. officinale* Rosc. var. *officinale*), red ginger (*Z. officinale* var. *rubrum*), and small white ginger (*Z. officinale* var. *amarum*) (Safitri et al., 2019). It is a tall annual herb that can grow as much as three feet tall. The stalk is encased in the sheathing bases of branched leaves. A stalk of yellow flowers with purple lips emerges from beneath the striking greenish-yellow bracts, giving the plant a club-like appearance. Rhizomes are 2–5 cm in width and 6–15 cm in length (Kumar et al., 2011). *Z. officinale* has been widely utilized in food as spices, flavoring agents and health supplements and widely used in traditional Chinese medicine (TCM), Ayurveda, and traditional Greco-Arab and Islamic medicine (Dhanik et al., 2017; Saad, 2022). *Z. officinale* has been utilized for numerous health disorders, including rheumatism, nausea, sore throat, common cold, fever, carminative, antipyretic, bronchitis, indigestion, gastrointestinal ailments, arthritis, pain, and appetite stimulant (Li et al., 2012; Baliga et al., 2013).

*Zingiber zerumbet* (L.) Smith is a perennial herb with tuberous roots that grows naturally in moist, shady areas of lowlands and slopes. It is mainly found in Asia and grows naturally in low-lying, moist, shady regions (Yob et al., 2011; Aji et al., 2022). The stems are erect, oblique, spherical, and covered in smooth leaf sheaths. The thin, 25–35 cm long leaves, which are occasionally purple beneath young shoots, have noticeably elevated midribs on the underside. A variety of ailments have been treated with wild ginger, such as migraine, nausea, morning sickness, motion sickness, hangovers, childhood worms, bruised skin, and wounds (Sahebkar, 2011; Haque et al., 2018).

*Zingiber cassumunar* Roxb. is found in Southeast Asia, particularly Indonesia and Thailand. It is a perennial herb composed of underground rhizomes (Chongmelaxme et al., 2017) and is found throughout tropical and subtropical Asia (Singh et al., 2015). Rhizomes have yellow flesh with a strong aroma. They are tuberous, cylindrical to ovoid, horizontal, creeping, irregular, profusely branched, and palmately, and laterally compressed. The leaves are simple, distichous, alternate, subsessile or shortly petiolate, lanceolate-oblong, and 3.5–5.5 × 18–35 cm long. The plant has been employed as an embrocation for many years (Lu et al., 2008). *Z. cassumunar* rhizome is popularly used as a kitchen spice and an ingredient of traditional medicines. In fact, people have empirically used it for various purposes, such as treating skin problems and as an immunomodulatory, anti-inflammatory, and anti-aging agent. In Thailand and other parts of Asia, *Z. cassumunar* is used both on its own and as an ingredient in herbal remedies for numerous ailments, such as inflammation, rheumatism, bruises, musculoskeletal pain, sprains and strains, asthma, coughs, respiratory problems, and wounds. It is also used as a mild laxative, carminative, anti-dysenteric agent, and mosquito repellent (Chongmelaxme et al., 2017).

*Alpinia galanga* Linn. was first found in Indonesia and is widely grown now in many Asian countries, including Indonesia, India, China, Sri Lanka, and Saudi Arabia. It is a perennial herb with underground rhizomes and small adventitious roots that can grow up to 3.5 cm. The surface of the rhizomes is reddish-brown, whereas their interior is brown and orange. Inner rhizome cuttings are distinguished by the presence of a black center surrounded by a larger and whiter layer on the outer rim. Galanga rhizomes have a strong aromatic odor and a spicy or pungent flavor (Kaushik, 2011; Verma and Sharma, 2022). Galangal rhizomes come in pink and light-yellow colors. Rhizomes from pink galangal measure 3 m in length and 8–10 cm in diameter. The pseudo-stem of the yellowish-white galangal rhizome is 1–1.5 m with a smaller diameter of 1–2 cm (Kaushik, 2011). Leaves are oblong-lanceolate, acute, and glabrous, green above and paler beneath, with somewhat callus white margins; sheaths are long and glabrous; and the ligule is short and rounded. Flowers are greenish white and densely bloom to 30 cm. The calyx is tubular and irregularly three-toothed (Verma and Sharma, 2022). It prefers warm areas with abundant sunlight, but it can also grow in shrubs, forests, and open areas (Ramanunny et al., 2022). The seeds of the galangal plant have many medicinal uses, including those for treating gastric disorders and cardiotonic lesions, as well as for their diuretic, antiplatelet, antifungal, and anti-tumor properties. For many common childhood illnesses, such as asthma, fever, dyspepsia, bronchitis, diabetes mellitus, and irritations, the galangal plant’s tuber is used as a cough therapy (Khairullah et al., 2020). Galangal rhizomes are used to treat aches and pains, colds, fever, diarrhea, heartburn or stomach pain, foul breath and body odor, severe thrush, sore throat, cough, and inflammation and to remove phlegm from bronchitis (Suciati and Maryati, 2023). The seed is used to treat emaciation and clean the mouth. It increases digestion and hunger while acting as a purgative. The rhizome is also commonly used as a spice. Flowers and young shoots are also consumed as vegetables or spices (Ramanunny et al., 2022; Verma and Sharma, 2022).

*Alpinia conchigera* Griff. is found in Vietnam, India, Thailand, Bangladesh, Indonesia, Malaysia, Cambodia, and Myanmar. The plant can grow up to 1.5-m tall. It is used in traditional medicine to treat inflammation-related diseases. Rheumatism is treated externally on the Malay Peninsula with a poultice of the cooked leaves or a mixture of the rhizomes and leaves. It is also consumed as a food condiment and to cure fungal diseases. In TCM, a boiling concoction of the rhizomes is used to treat dyspepsia, whereas a pungent paste prepared from the rhizomes is used for insect bites (Aziz et al., 2013). *A. conchigera* is also traditionally used to treat ailments such as the common cold, stomachache, bloating, digestive issues, and joint discomfort (Hanh and Quoc Binh., 2014). Crushed leaves are often given postpartum. Hot aqueous infusion of *A. conchigera* rhizomes is believed to be beneficial to women during confinement to make the body feel warm, enhance blood circulation, stimulate uterine contraction, expel wind, avoid fits, and function as a laxative (Jamal et al., 2011).

*Alpinia oxyphylla* Miq. is wildly distributed in China in the provinces of Hainan, Guangdong, Fujian, Yunnan, and Guangxi. The plant is a clustered perennial herb that can grow up to 3 m. Leaves are lanceolate, 25–35 cm long, 3–6 cm wide, apex tail-tipped, with nearly round base, margin with small deciduous bristles, short petioles, and membranous ligules, which are two-lobed, 1–2 cm long, and sparsely covered with light brown hairs. The fresh capsules are spherical, spindle-shaped when dry, 1.5–2 cm long, pubescent, with bulging vascular bundle lines, and maturely green-yellow or cream. The seeds are numerous, irregularly round, and flat with a pale-yellow aril (Zhang et al., 2018). *A. oxyphylla* is widely cultivated as an important industrial crop in Southern China and has been utilized for hundreds of years as a medicinal ingredient. It has therapeutic properties, including a neuroprotective effect (Wang et al., 2015; Qiu et al., 2023; Qiu et al., 2023), sedation, and hypnosis; improves learning and memory abilities, antioxidant, anti-tumor, anti-inflammatory (Qiu et al., 2023); and has other pharmacological activities including intestinal disorders, abdominal pain, diarrhea, dementia-related conditions, as well as enhancing cognitive performance and being aphrodisiac and anti-polyuric (Wang et al., 2015).

*Alpinia officinarum* Hance was first found in Southern China and is distributed over quite a wide area of Vietnam, Thailand, Singapore, Philippines, Myanmar, Malaysia, Indonesia, China, Cambodia, and Bangladesh (Tungmunnithum et al., 2020). It grows well at low to mid-elevations in the forests of tropical and subtropical regions. It is widely cultivated in Southeast Asia (Zou et al., 2016). The rhizome is dark reddish brown with a potent aromatic odor (Dixit et al., 2012), the leaves are lineolate acuminate, and the flowers are white showy racemes (Basri et al., 2017). The rhizome is widely used to treat a variety of ailments, including the common cold, rheumatism, discomfort, bronchial catarrh, bad breath, ulcers, whooping cough in children, throat infections, stomachache, cancer, viral diseases, inflammation, microbial infection, cardiovascular diseases, and type 2 diabetes mellitus (Mukherjee et al., 2021; Bitari et al., 2023a; Ahmad et al., 2023; Zhang et al., 2023).

*Alpinia katsumadai* Hayata is distributed in many Southeast Asian countries (Li et al., 2012). The seed is composed of sub-spheroidal seeds ranging in diameter from 15 to 27 mm. When cut in half longitudinally along the raphe, the morphology of the seed is obliquely cordate. The endosperm is an off-white hue. Seeds possess a distinctive aroma and a pungent, slightly bitter flavor (Nam and Seo, 2012). TCM uses *A. katsumadai* to heal emesis and gastric disorders (Groblacher et al., 2012). The seeds have been utilized as an antioxidant and stomachic (Park et al., 2020).

*Alpinia purpurata* (Viell.) K. Schum*.* originates from the Pacific islands and is a popular plant in Brazil and India (Chan and Wong, 2015). It is also known as red ginger. It is a plant of middling size and can grow up to 2 m. The leaves are oblong in shape, alternate in arrangement, and sessile and have a pointed tip. The leafy shoots of the plant mature into attractive inflorescences, which are spikes that stand upright and are covered in showy bracts in either red or pink. Rhizomes and the stalks of the leaves have a fragrant quality (Chan and Wong, 2015). The plant is used to manufacture numerous useful products, such as foods, spices, medicines, perfumes, dyes, and fiber papers (Delira et al., 2015). In India, rhizomes are utilized in ethnomedicine to heal headache, sore throat, rheumatism, and renal infection and to improve voice, taste, and appetite. In Venezuela, the hot water infusion of inflorescences of *A. purpurata* is used to treat cough symptom (Palanirajan et al., 2022). The lectins from *A. purpurata* inflorescence showed antileukemia potential (Brito et al., 2023). Previous studies showed the potential effects of *A. purpurata* rhizome against fungal infection (Azizah et al., 2022) and cancer (Palanirajan et al., 2022).

*Alpinia zerumbet* (Pers.) Burtt. Et Smith is native to East Asia and found in subtropical and tropical countries, including India, Taiwan, Brazil, Japan, and Malaysia (Kawai et al., 2021). *A. zerumbet* is a fragrant perennial herb that spreads by rhizomes and has a short stalk that can grow up to 3 m. The leaves are fragrant and lanceolate and have a consistency similar to the coriaceous material. The flowers are shaped like funnels (Junior et al., 2017). The flowers, leaves, and rhizomes all have their own unique scents. *A. zerumbet* is utilized in traditional medicine to treat hypertension, colds, and inflammation and as an antispasmodic (Kawai et al., 2021). The plant has been reported for its antiviral (Chen et al., 2014; Morimoto et al., 2022) and antifungal activities (Okazaki et al., 2023).

## 4 Phytochemistry

The main groups of phytochemicals in *Z. officinale* are terpenoids, flavonoids, phenolic compounds, glycosides, alkaloids, saponins, and sterols. The volatile oil is mostly made up of monoterpenes and sesquiterpenes. These include β-phellandrene, camphene, limonene, cineole, geraniol, geranyl acetate, α-zingiberene, zingiberol, curcumene, borneol, and β-elemene (Dhanik et al., 2017). The major components of ginger extract are gingerols and shogaols, including 6-shogaol, 8-shogaol, 10-shogaol, 10-gingerol, 8-gingerol, and 6-gingerol (Mao et al., 2019). Other phytochemical constituents found in ginger are zingerone (Schadich et al., 2016; Ji et al., 2017; Murti et al., 2022), 6-dehydrogingerdione, quercetin, gingerenone-A, zingerone, geranial, and eugenol (Singh et al., 2008; Mao et al., 2019). Fresh organically grown ginger analyzed by GC-MS was found to contain shogaols, gingerols, gingerdiols, 3-dihydroshogaols, acetyl derivatives of gingerols, methyl ether derivatives of shogaols, mono- and diacetyl derivatives of gingerdiols, diarylheptanoids, 1-dehydrogingerdiones, paradols, and dihydroparadols (Jolad et al., 2004). Catechol, p-hydroxy benzoic acid, chlorogenic acid, vanillic acid, caffeic acid, p-coumaric acid, benzoic acid, ferulic acid, and rosmarinic acid were among the phenolic and flavonoid constituents found in ginger peel extract examined by HPLC (Shalaby et al., 2023). According to GC and GC-MS analyses, the essential oil of ginger contained α-pinene, β-pinene, α-phellandrene, α-terpineol, β-elemene, α-zingiberene, α-(E,E)-farnesene, δ-cadinene, β-sesquiphellandrene, β-eudesmol, camphene, methyl-5-hepten-2-one, myrcene, limonene, 1,8-cineole, terpinolene, linalool, borneol, neral, geraniol, bornyl acetate, thymol, germacrene D, ar-curcumene, trans-muurola-4(14)5-diene, germacrene B, trans-nerolidol, ar-turmerone, α-turmerone, and β-turmerone (Guerrini et al., 2023).

*Z. zerumbet* is an abundant source of several chemical metabolites, including polyphenols, alkaloids, and terpenes (Koga et al., 2016). Spectrophotometric analysis of the rhizomes, leaves, and stems of *Z. zerumbet* identified phenolic acids (gallic acid, cinnamic acid, caffeic acid, and ferulic acid) and flavonoids (kaempferol, catechin, rutin, quercetin, rutin, myricetin, and luteolin) (Ghasemzadeh et al., 2016). The rhizome oil contained a very high concentration of zerumbone (69.9%). The other compounds, such as α-humulene, camphene, caryophyllene oxide, and humulene epoxide II, were present in appreciable amounts (Rana et al., 2012). HPLC-DAD-ESI-MS analysis of the rhizome active fractions revealed the presence of kaempferol glycosides, which include kaempferol 3-O-(2″-O-acetyl) rhamnoside, kaempferol 3-O-rhamnoside, kaempferol 3-O-(4″-O-acetyl) rhamnoside, kaempferol 3-O-(2″,4″-O-diacetyl) rhamnoside, kaempferol 3-O-(3″-O-acetyl) rhamnoside, and kaempferol 3-O-(3″,4″-O-diacetyl) rhamnoside (Ruslay et al., 2007). Twenty-nine compounds were found in the leaf oil by GC-MS, where zerumbone, α-caryophyllene, and camphene were the major constituents. In another study, the rhizome oil was found to contain 30 compounds where zerumbone, α-caryophyllene, and 1,5,5,8-tetramethyl-12-oxabicyclo[9.1.0]dodeca-3,7-diene were the main components (Bhuiyan et al., 2008). Zerumbone was identified as the major compound contributing to the gastroprotective activity of *Z. zerumbet* (Murti et al., 2022).

*Z. cassumunar* is rich in phenylbutanoids or cassumunarins, which have a crucial function as antioxidants in delivering photoprotective action to UV-B-exposed skin. The rhizome extract of the plant contained terpinen-4-ol, α-pinene, β-pinene, cassumunarin A, cassumunarin B, cassumunarin C, phenyl butanoic dimers, p-cymene, myrcene, limonene, α-terpinene, sabinene, and terpinolene (Singh et al., 2015). *Z. cassumunar* also contains terpinen-4-ol, phenyl butanoic dimer, myrcene, and sabinene (Han et al., 2021). GC-MS analysis of the essential oil of *Z. cassumunar* revealed monocyclic monoterpenoids as the major components, and sesquiterpenes were in small amounts. They were α-thujene, sabinene, α-pinene, α-terpinene, myrcene, benzene, γ-terpinene, α-terpinolene, terpinen-4-ol, β-sesquiphellandrene, and 1,2-dimethyl-6-nitroindolizine (Mektrirat et al., 2020). The phytochemical study on *Z. cassumunar* rhizome identified β-sitosterol (E)-4-(3′,4′-dim ethoxyphenyl) but-3-en-1-ol,3,4-dimethoxybenzoic acid, cis-3-(3′,4′-dimethoxyphenyl)-4-[(E)-3‴,4‴- dimethoxystyryl]cyclo-hex1-ene, and 8-(13,14-dimethoxyphenyl)-2-methoxynaphto-1,4-quinone (Zulkhairi et al., 2017).

*A. galanga* contains high amounts of phenolic compounds, including phenolic acids and flavonoids. The major constituents of the rhizome include galangoisoflavonoid, diglucosyl caprate, p-coumaryl diacetate, methyleugenol, trans-p-acetoxycinnamyl alcohol, galangin, 1′-acetoxyeugenol acetate, β-sitosterol, trans-3, 4-dimethoxycinnamyl alcohol, trans-p-coumaryl alcohol, acetoxychavicol acetate (ACA), hydroxychavicol acetate, and 1′S-1′-acetoxychavicol acetate (ACE) (Kaushik, 2011). It was also reported that 1′-acetoxychavicol acetate (E)-8β, 17-epoxylabd-12-ene-15, 16-dial, 1, 7-bis(4-hydroxyphenyl)-1,4,6-heptatrien-3-one, 1′S-1′-acetoxyeugenol acetate *p*-hydroxycinnamaldehyde, and bisdemethoxycurcumin trans-p-acetoxycinnamyl alcohol were present in the plant (Chouni and Paul, 2018). The components of *A. galanga* essential oil were analyzed using the head space solid phase microextraction GC-MS. Forty-three volatile chemicals were identified, which were made up of alcohols, alkenes, esters, and other minor compounds. Fresh *A. galanga* samples were found to contain 29 chemical constituents, including 3 alcohols, 17 alkenes, 7 esters, and 2 others (Ge et al., 2022). (R)-4-(1-Methoxypropyl)phenol; (S)-3-(4-hydroxy-3-methoxyphenyl) propane-1,2-diyldiacetate; (R)-3-(4-hydroxy-3-methoxyphenyl)propane-1,2-diyldiacetate; and 3′-demethoxycrataegusanoid E were found in *A. galanga* fruits (Liu et al., 2023).

*A. purpurata* inflorescences were reported to contain lectin (carbohydrate-binding protein). Forty-two components with β-caryophyllene, β-pinene, and α-pinene as the main constituents were identified in *A. purpurata* essential oil by GC-MS (Santos et al., 2012). Another GC-MS analysis identified 30 compounds, which were mainly monoterpenes and sesquiterpenes. The main components of the oil were trans-caryophyllene, β-pinene, α-pinene, 7-epi-α-selinene, and camphene (Delira et al., 2015). HPLC analysis demonstrated that rutin and kaempferol-3-O-glucuronide were found in greater quantities in ethyl acetate and butanol extracts of *A. purpurata* dried leaves (Victorio et al., 2009). Phytochemical analysis of *A. purpurata* identified 6-gingerol, 8-gingerol, 10-gingerol, 4-shogaol, 6-shogaol, 10-shogaol, α-pinene, 1,8-cineole, β-pinene, and (*E*)-methylcinnamate as major constituents (Shimoda et al., 2007).

The rhizome oil of *A. conchigera* contained eucalyptol as the primary component (25.85%), as determined by GC-MS analysis. The other compounds found in the oil were chavicol, caryophyllene, α-pinene, camphene, 4-terpineol, γ-terpinene, eugenyl acetate, 4-(2, 6, 6-trimethyl-1-cyclohexan-1-yl), and 3-buten-2-ol (Bhuiyan et al., 2008). 1′S-1′-hydroxychavicol acetate, 1′S-1′-acetoxychavicol acetate (AEA), trans-p-coumaryl diacetate, p-hydroxycinnamyl acetate, p-hydroxybenzaldehyde, β-sitosterol, and stigmasterol have been isolated from *A. conchigera* rhizome (Taib et al., 2020). Column chromatography separation of the n-hexane and dichloromethane extracts of pseudostems and rhizomes of *A. conchigera* yielded caryophyllene oxide, p-hydroxycinna maldehyde, p-hydroxycinnamyl acetate, 1′-hydroxychavicol acetate, trans-p-coumaryl diacetate, chavicol acetate, 1′S-1′-acetoxychavicol acetate, 4-hydroxybenzaldehyde, and a mixture of β-sitosterol and stigmasterol (Aziz et al., 2013).

*A. officinarum* rhizome ethanol extract was reported to be rich in carbohydrates (20.25%). Other components, such as protein, phenolic, tannins, flavonoids, and lipids, were present in the range from 2.79% to 18.26% (Alasmary et al., 2019). From the chloroform extract of *A. officinarum*, four diarylheptanoids: (5S)-5- hydroxy-7-(3, 4-dihydroxyphenyl)-1-phenyl-3-heptanone, (5R)-5-hydroxy-7-(3- methoxy-4, 5-dihydroxyphenyl)-1-phenyl-3-heptanone, 7-(3,4-dihydroxyphenyl)-1-(4-hydroxy-3-methoxyph enyl)-4-en-3-heptanone, and (5R)-5-hydroxy-1-(3,4-dihydroxy phenyl)-7-(4-hydroxy-3-methoxyphenyl)-3-heptanone were isolated (An et al., 2008). Diarylheptanoids found in the ethanol extract of *A. officinarum* rhizomes were identified as 1, 7- diphenyl-5-heptene-3-one, 4-phenethyl-1, 7-diphenyl-1-heptene-3, 5-dione, and 7-(4″, 5″-dihydroxy-3″-methoxyphenyl)-1-phenyl -4-heptene-3-one (Zhang et al., 2010).

Four diarylheptanoids, two flavonoids, and one sterol were found in *A. officinarum* rhizomes. The compounds identified were galangin, kaempferide, β-sitosterol, 1-(4-hydroxy-3-methoxyphenyl)-7-phenyl-3,5-heptanediol, 5-hydroxy-7-(4-hydroxyphenyl)-1-phenyl-3-hepta none, 3-*O*-β-D-6-palmitoylgluco side (3*R*,5*R*)-1-(4-hydroxyphenyl)-7-phenyl-3,5-heptanediol, (5R)-5-hydroxy-7-(4-hydroxy-3-methoxyphenyl)-1-(4-hydroxyphenyl)-3-heptanone, and 5-hy droxy-1-(4-hydroxy-3-methoxyphenyl)-7-(4-hydroxyphenyl)-3-hept anone (Shin et al., 2002). Diarylheptanoids and galangin were suggested to be mainly responsible for the antiproliferative activity of the plant toward human glioblastoma cancer cells (Liu et al., 2014).

*A. katsumadai* was reported to contain nootkatone, yakuchinone, kaempferide, tectochrysin, apigenin-4′,7′-dimethylether, chrysin, oxyphyllacinol, izalpinin, steroid and their glycosides, and volatile oils (Qing et al., 2012). Pinocembrin (dihydrochrysin), (5R)-trans-1,7-diphenyl-5-hydroxyhept-6-en-3-one, trans, trans-1,7-diphenylhepta-4,6-dien-3-one, and (3S,5S)-trans-1,7-diphenylhept-1-ene-3,5-diol have been isolated from *A. katsumadai* (Gröblacher et al., 2012). An isocoumarin (3R)-5,6,7-trihydroxy-3-isopropyl-3-methylisochroman-1-one from *A. katsumadai* seeds demonstrated a neuroprotective effect on oxidative damage in PC12 cells stimulated by 1-methyl-4-phenylpyridinium (Chen et al., 2016). The seeds contained (1R,3R,4S)-1-(4′-methyl-phenyl)-3,4-dihydro-3,4-dimethyl-1H-2-b enzopyran-5,6,8-triol and (3R)-5,7-dihydroxy-3-isopropyl-3-methylisochroman-1-one. The latter exhibited significant suppression of OVA-induced allergic airway inflammation with an associated decrease in the production of IgE and Th2 cytokines (Wang et al., 2017).

*A. oxyphylla* has numerous active ingredients with a wide range of pharmacological effects (Chen et al., 2014; Zhang et al., 2015). 5-Hydroxymethylfurfural (5-HMF) was the most active component of *A. oxyphylla* ethanol extract that demonstrated memory-enhancing effects against Alzheimer’s disease (Liu et al., 2014). Nootkatone identified in *A. oxyphylla* was found to possess anti-diarrhea (Zhang et al., 2013) and insecticidal effects (Miyazawa et al., 2000). *A. oxyphylla* was also found to contain tectochrysin and yakuchinone A (Zhang et al., 2013). *A. oxyphylla* also contains yakuchinone A (1-[4-hydroxy-3-methoxyphenyl]-7-phenyl-3-heptanone) and yakuchinone B (1-[4-hydroxy-3-methoxyphenyl]-7-phenylhept-1-en-3-one) reported to stimulate apoptosis in HL-60 cells (Jha et al., 2015). Oxyphyllenones A and B and oxyphyllenodiols A and B were identified in the methanol extract of *A. oxyphylla* kernels (Muraoka et al., 2001). Phytochemical analysis of *A. oxyphylla* fruits resulted in the isolation and identification of 40 structurally varied sesquiterpenoids, including 17 new eudesmane sesquiterpenoids and 23 known analogs. Orthorhombic and neoxyphyllanene were unique rearranged eudesmane sesquiterpenoids found among the isolates (Dong et al., 2023). Mass spectrometry and NMR were used to identify 15 chemicals found in an ethyl acetate extract of *A. oxyphylla*: 3,5-dihydroxy-7,4-dimethoxyflavone; 1-(3″-5″-dihyd roxy-4″-methoxy-phenyl)-7-phenyl-3-heptanone; chrysin; tectoch rysin; kaempferol; baicalein; wogonin; myricetin; yakuchinone A; mangiferin; protocatechuic acid; vanillic acid; teuhetenone A; oleanolic acid; and β-sitosterol (Xu et al., 2023). *A. oxyphylla* has an abundance of (E)-labda-8(17),12-diene-15,16-dial, and the main volatiles of *A. oxyphylla* were L-β-pinene, trans-sabinene hydrate, and cyclofenchene (Peng et al., 2022).

Alpinetin, cardamomin, and other flavonoids have been isolated from *A. katsumadai* and *A. zerumbet* seeds and rhizomes (He et al., 2005). According to recent studies, the leaf extracts of *A. zerumbet* and *A. purpurata* were found to contain rutin, kaempferol-3-O-glucuronide, and diarylheptanoids (Victório et al., 2007; Victório et al., 2009b; Victório C. P. et al., 2009; Victório et al., 2009d; Victório et al., 2009; Victório et al., 2010a). LC-MS analysis showed the presence of six kavalactone derivatives in the methanol extracts of the pericarps, placenta, and leaves of *A. zerumbet*. One of these compounds was found to be a new asymmetrical cyclobutane dimer of 5,6-dehydrokawain (Nishidono et al., 2020).

## 5 Immunomodulating effects of *Zingiber* species

**FIGURE 3:**
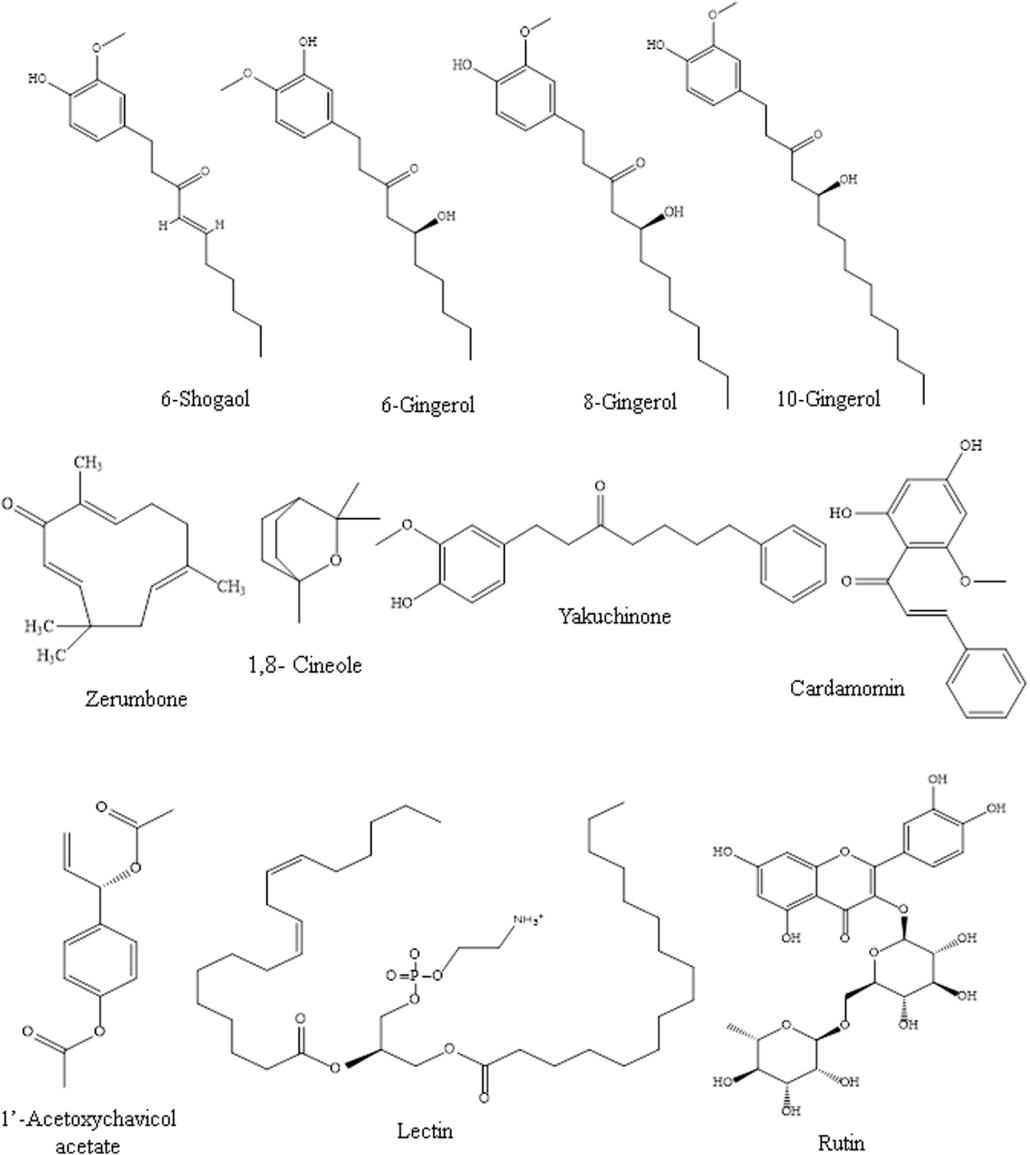
Chemical structures of the bioactive metabolites of *Zingiber* and *Alpinia* species with strong immunomodulating activities.

Many *Zingiber* species, especially *Z. officinale*, *Z. cassumunar*, and *Z. zerumbet*, have been evaluated for their immunomodulating effects, particularly via signaling pathways. Most of the studies on their immunomodulating activities were on the plant crude extracts, and the bioactive metabolites of some of the extracts, particularly gingerols, shogaols, and zerumbone, responsible for the immunomodulating activities, have been reported. Table 1 shows the modulatory effects and mechanisms of the extracts of *Z. zerumbet*, *Z. officinale*, and *Z. cassumunar* on the immune system. The modulatory effects and mechanisms of bioactive secondary metabolites of the *Zingiber* species on the immune system are shown in Table 2. Figure 3 depicts the chemical structures of the bioactive metabolites of *Zingiber* and *Alpinia* species with strong immunomodulating activities.

**TABLE 1:** Immunomodulatory effects and mechanisms of the extracts of *Zingiber* and *Alpinia* species.

Species	Subject	Study design/method	Preparation	Immunomodulatory activity	Modulation	Parameter/mediator affected	Reference
*Zingiber officinale*	Osteoarthritis patients	Clinical study	Essential oil	Cytokine release	Suppression	TNF-α, IL-1β	Mozaffari-khosravi et al. (2016)
	Autoimmune encephalomyelitis mice	*In vivo*	Ethanol extract	Cytokine release	Suppression	IL-33	Jafarzadeh et al. (2014a)
	Fibromyalgia mouse	*In vivo*	Dried powder	Proinflammatory Mediators	Suppression	TXB2, PGE2, IL-1β, and NO	Montserrat-de paz et al. (2018)
	Osteoarthritis rats	*In vivo*	Ethanol extract	Cytokine Release	Suppression	IL-1β	Aborehab et al. (2017)
	Human T lymphocytes	*In vitro*	Methanol extract	Inflammatory cytokines	Stimulation	IL-8, TNF-α, and IFN-γ	Schoenknecht et al. (2016)
	Pulmonary inflammation model mouse	*In vivo*	Aqueous extract	Th1 and Th2 cell activity	Suppression	Th1 and Th2 cell	Ahui et al. (2008)
				Cytokine release	Suppression	IgE	
	Human lymphocytes	*In vitro*	Powder	Cytokine release	Suppression	IL-2 and IL-10	Wilasrusmee et al. (2002)
	Encephalomyelitis model mice	*In vivo*	Ethanol extract	Cytokine release	Suppression	IL-23 and IL-17	Jafarzadeh et al. (2015a)
	OVA-induced allergic rhinitis in mice	*In vitro*	Oral supplement	Cytokine release	Suppression	Th1 and Th2 cell	Kawamoto et al. (2016)
	LPS-activated BV2 microglia	*In vitro*	Ethanolic extract	Cytokine release	Suppression	IL-1β, IL-6, and TNF-α	Ho et al. (2013)
	Tumor mouse model	*In vivo*	Ginger polysaccharide (UGP1)	Cytokine release	Stimulation	TNF-α, IL-2, and IL-6	Qian et al. (2023)
	Tumor mouse model	*In vivo*	Ginger polysaccharide (UGP1)	Cytokine release	Suppression	TGF-β and bFGF	Qian et al. (2023)
	BALF of BALB/c mice model of ovalbumin-induced allergic asthma	*In vivo*	Ethanol extract and aqueous extract	Total and differential count of granulocytes	Suppression	Eosinophils and neutrophils	Harun and Mohamad (2022); Khan et al. (2015)
	BALF of BALB/c mice model of ovalbumin-induced allergic asthma	*In vivo*	Ethanol extract and aqueous extract	Cytokine release	Suppression	IL-4, IL-5, and IgE	Harun and Mohamad (2022); Khan et al. (2015)
	Community-acquired pneumonia (CAP) patients	Clinical study	*Z. officinale* capsule 300 mg	Serum inflammatory markers	Suppression	Procalcitonin and NLR	Reviono et al. (2023)
	Peritoneal adhesion model of male Wistar rats	*In vivo*	Ethanol extract	Cytokine release	Suppression	IL-6, TNF-α, TGF-β1, and IL-10	Yahyazadeh et al. (2023)
*Zingiber zerumbet*	Human	*In vitro*	Ethanol extract	CD18 expression	Suppression	CD18	Akhtar et al. (2019)
	Neutrophil cell						
	Mice	*In vivo*	Ethanol extract	CD11b/CD18 expression	Suppression	CD11b/CD18	Ghazalee, et al. (2019)
*Zingiber cassumunar*	Mice macrophages	*In vitro*	Ethanol extract	Cytokine release	Stimulation	IL-10 and IL-l4	Mahfudh et al. (2020a)
	Mice macrophages	*In vitro*	Ethyl acetate fraction	ROS enhance	Stimulation	CD36	Nurkhasanah and Sofyan (2019)
	Mice macrophages	*In vivo*	Ethyl acetate fraction	Cytokine release	Stimulation	IL-10 and IL-l4	Nurkhasanah and Sofyan (2019)
	Mice macrophages	*In vitro*	Chloroform fraction	Phagocytic effects	Stimulation	Phagocytes	Ramadhan et al. (2020)
	Adult asthmatic patients	Clinical study	*Z. cassumunar* capsule	Bronchial hyperresponsiveness	Suppression	Fractional exhaled nitric oxide	Dulpinijthamma et al. (2023)
*Alpinia galanga*	HaCaT keratinocyte cell	*In vitro*	Ethanol extract	Inflammatory cytokines	Suppression	NF-κB signaling	Saelee et al. (2011)
					Stimulation	TNF-α	
	LPS-stimulated RAW 264.7 cell	*In vitro*	Ethanol extract	Cytokines release	Suppression	IL-6 and TNF-α	George et al. (2021)
	Mice	*In vivo*	Flavonoid fraction	Lymphocyte formation	Stimulation	Lymphocyte	Jain et al. (2012)
	Mice	*In vivo*	Flavonoid fraction	T-cell proliferation	Stimulation	T-cell proliferation	Jain et al. (2012)
*Alpinia conchigera*	LPS-induced RAW 264.7 cells	*In vitro*	Isolate	NF-κB activation	Suppression	NF-κB	Lee et al. (2006)
	MCF-7 cell	*In vitro*	Isolate	Dysregulation of NF-κB pathway	Suppression	NF-κB	In et al. (2011)
*Alpinia katsumadai*	OVA-induced asthma mouse model	*In vitro*	Ethanol extract	Cytokine release	Suppression	Th2-type cell	Lee et al. (2010)
						IgE and IgG2a	
	Mouse paw edema	*In vivo*	Powder	Cytokine release	Suppression	NO, TNF-α, IL-1β, and IL-6	Li et al. (2015)
	Mouse paw edema	*In vivo*	Powder	iNOS and COX-2 expressions	Suppression	iNOS and COX-2	Li et al. (2015)
	Mouse paw edema	*In vivo*	Powder	NF-κB activation	Suppression	NF-κB	Li et al. (2015)
	Mouse paw edema	*In vivo*	Powder	ERK1/2, p38, and JNK/SAPK level	Suppression	ERK1/2, p38, and JNK/SAPK	Li et al. (2015)
	Allergic airway inflammation induced by OVA in mice	*In vitro*	Isolate	Inflammatory cytokines	Suppression	Th2 cell and IgE	Wang et al. (2017)
*Alpinia oxyphylla*	Monosodium iodoacetate (MIA)-induced rat model	*In vitro*	Ethanol extract	Cytokine release	Suppression	IL-1β, IL-6, and TNF-α	Lee et al. (2019)
	LPS-treated RAW 264.7 cell	*In vitro*	Ethanol extract	Cytokine release	Stimulation	ERK, JNK, and MAPK	Lee et al. (2019)
	Lipopolysaccharide-induced mice model of Alzheimer’s disease	*In vivo*	Petroleum ether extract *of A. oxyphylla* fructus	Cytokine release	Suppression	IL-1β	Guo et al. (2022); Wang et al. (2018)
*Alpinia zerumbet*	LPS-induced HAEC	*In vitro*	Essential oils	Inflammatory cytokines	Suppression	TLR4/NF-κB	Xiao et al. (2020)
	Endothelial cell	*In vitro*	Essential oil	Inflammatory cytokines	Suppression	NF-κB	Huang et al. (2017)
	Endothelial cell	*In vitro*	Essential oil	Inflammatory cytokines	Suppression	TNF-α and IL-8	Huang et al. (2017)
*Alpinia officinarum*	Macrophage cell and human peripheral blood mononuclear cells (PBMCs)	*In vitro*	Isolate	Cytokine release	Suppression	IL-1β	Yadav et al. (2003)
	Macrophage cell and human peripheral blood mononuclear cells (PBMCs)	*In vitro*	Isolate	Cytokine release	Suppression	TNF-α	Yadav et al. (2003)

**TABLE 2:** Immunomodulatory effects and mechanisms of bioactive metabolites of *Zingiber* and *Alpinia* species.

Main compound	Species	Subject	Study design	Immunomodulatory activity	Modulation	Parameter/mediators affected	Reference
6-Shogaol	*Z. officinale*	Mast cell-mediated allergic	*In vitro*	Reduced release of proinflammatory cytokines	Suppression	NF-kB and phosphorylation of JNK	Van Breemen et al. (2011)
		Human mast cells (HMC-1)	*In vitro*	Inhibited production of TNF-α and IL-6	Suppression	TNF-α and IL-6	Sohn et al. (2013)
		Human mast cells (HMC-1)	*In vitro*	Inhibited activation of NF-kB p65 via stabilizing IkB-α	Suppression	TNF-α, IL-6, and IL-8	Sohn et al. (2013)
		THP-1 macrophages	*In vitro*	Attenuated canonical NLRP3 inflammasome-mediated IL-1β secretion	Suppression	Pro-IL-1β and NLRP3	Ho & Chang (2018)
6-Gingerol	*Z. officinale*	Ulcerative colitis mice model	*In vivo*	Decreased Il-17 level	Suppression	Il-17	Sheng et al. (2020)
		Ulcerative colitis mice model	*In vivo*	Increased IL-10	Stimulation	IL-10	Sheng et al. (2020)
		Ulcerative colitis mice model	*In vivo*	Suppressed phosphorylation level of IkB-α and p65	Suppression	Regulating NF-κB signaling pathway	Sheng et al. (2020)
		Spleen cells from a BALB/c mouse	*In vitro*	Inhibited cytokine production of Th1 and Th2 from T lymphocytes	Suppression	Reduction of allergic rhinitis symptoms	Kawamoto, et al. (2016)
		Mononuclear cells of experimental autoimmune encephalomyelitis in mice brain	*In vivo*	Decreased the inflammatory cell infiltration from the peripheral blood into the CNS	Suppression	IL-17	Harun and Mohamad (2022); Han et al. (2019)
		Proximal colon	*In vivo*	Decreased inflammatory cytokines	Suppression	IL-1β, TNF-α, and IL-6	Harun and Mohamad (2022); Cakir et al. (2018)
6-Gingerol, 8-gingerol, and 10-gingerol	*Z. officinale*	Human T cell model	*In vitro*	Enhanced levels of inflammatory cytokines such as IL-8 and TNF-α	Stimulation	IL-8 and TNF-α	Schoenknecht et al. (2016)
Zerumbone	*Z. zerumbet*	LPS	*In vitro*	Decreased in CD18 expression in polymorphonuclear neutrophils	Suppression	CD18 expression	Akhtar et al. (2019)
		Splenocytes	*In vitro*	Suppressed proliferation of T and B lymphocytes and inhibited the release of Th1 and Th2 cytokines	Suppression	CD8^+^ and CD4^+^	Jantan et al. (2019)
		LPS-induced human macrophages	*In vitro*	Inhibited proinflammatory mediators (COX-2, PGE2 IL-1β, and TNF-α)	Suppression	COX-2, PGE2 IL-1β, and TNF-α	Haque et al. (2018)
		Spleens	*In vitro*	Increased cytotoxic effect on T cells, natural killer T cells, and helper T cells	Suppression	Suppressed IL-1β and IL-6 levels and increased the serum IL-2 and IFN-γ levels	Mohamad et al. (2015)
		LPS- and IFN-γ-induced RAW 264.7 mouse macrophages	*In vitro*	Suppressed protein expressions	Suppression	iNOS synthase and COX-2	Nuzzo et al. (2022); Murakami et al. (2002)
		Methicillin-resistance *Staphylococcus aureus*	*In vitro*	Suppressed ROS production	Suppression	ROS	Albaayit et al. (2022)
		Colorectal cancer- BALB/c mice	*In vivo*	Antiangiogenic action	Stimulation	miR-34a	Nobari et al. (2023)
		Human PMNs	*In vitro*	Suppressed ROS production	Suppression	ROS	Ibanez et al. (2023); Akhtar et al. (2019)
1′-Acetoxychavicol acetate (ACA)	*Alpinia galanga*	OVA-induced asthma animal models	*In vivo*	Suppression of eosinophil infiltration and a reduction in IgE level	Suppression	IgE level	Seo et al. (2013)
				Inhibited release of T-helper type-1 cytokines IL-12 and interferon and decreased the expression of T-helper type 2 cytokines, such as IL-4 and IL-13	Suppression	T-helper type-1 cytokines IL-12 and interferon-α, the expression of T-helper type 2 cytokines, such as IL-4 and IL-13	
				Suppressed cytotoxic T cell	Suppression	CD8 and CD4 Th cells	
				Suppressed expression of Th1/2 cells	Suppression	Th2 cytokine (IL-4, IL-6, and IL-13) and Th1 cytokine (IL-12α, IFN-γ	
Cardamomin	*A. katsumadai*	Endothelial cells	*In vitro*	Decreased NO, TNF-α, IL-1β, and IL-6 levels	Suppression	Transmigration of leukocytes	Li et al. (2015)
		Mouse paw	*In vivo*	Decreased ERK1/2, p38, and JNK/SAPK level	Suppression	ERK1/2, p38, and JNK/SAPK level	
	*A. conchigera*	RAW 264.7 cells	*In vitro*	Reduced expression of the NF- B reporter gene that was activated by LPS or tumor necrosis factor (TNF)	Suppression	TNF-α and NO	
	*A. conchigera*	LPS/IFN-γ-induced RAW 264.7 cells	*In vitro*	Inhibited PGE2 and NO production	Suppression	TNF-α production	Gonçalves et al. (2014)
	-	Rheumatoid arthritis rat model	*In vivo*	Inhibition in TNF-α, IL-1β, and IL-6 levels	Suppression	TNF-α, IL-1β, and IL-6 levels	Voon et al. (2017)
Yakuchinone	*Alpinia oxyphylla*	LPS-activated RAW 264.7 cells	*In vitro*	Reduced expression of iNOS and suppressed mRNA expression	Suppression	iNOS protein and mRNA expression	Lee et al. (2006)
		12-O-Tetradecanoylphorbol-13-acetate (TPA)-induced ear edema of mouse	*In vivo*	Inhibited TNF-α production	Suppression	TNF-α	Chun et al. (2002)
		Mouse lymphoma EL4 cells	*In vitro*	Reduced IL-17 production	Suppression	IL-17 levels	Huang et al. (2019)
1,8-Cineole	Zingiberaceae family	LPS-induced alveolar macrophages	*In vitro*	Reduced levels of inflammatory mediators (TNF-α, IL-1α, IL-1β, and NO	Suppression	TNF-α, IL-1α, IL-1β, and NO	Yadav and Chandra (2017)
		Bronchoalveolar lavage fluid	*In vitro*	Reduced NF-κB expression, TLR4, neutrophils, and macrophages	Suppression	NF-κB, TLR4, neutrophils, and macrophages	Zhao et al. (2013)
		Hyperammonemia rat animal model	*In vivo*	Inhibit ROS production	Suppression	ROS	Bahrami et al. (2023)
		Hyperammonemia rat animal model	*In vivo*	Inhibited cytokine release	Suppression	IL-6, IL-1β	Bahrami et al. (2023)
		Chronic rhinosinusitis patients	Clinical study	Inhibited T-cell proliferation	Suppression ↓	T-cell proliferation	Polasky et al. (2021)
Lectin	*Alpinia purpurata*	Murine model of schistosomiasis	*In vivo*	Increased globulin and creatine kinase MB (CK-MB) isoenzyme	Stimulation	Globulin and creatine kinase MB (CK-MB)	Brito et al. (2017)
		Mouse peritoneal macrophages	*In vitro*	Reduced NO production and inhibited proliferation of mononuclear cell	Suppression	NO production	Ditamo et al. (2016)
Rutin	Zingiberaceae family	Docking	*In silico*	Docked with the active sites of TNF-α, IL-1, IL-6, and NO and revealed positive interactions with these targets	Suppression	TNF-α, IL-1, IL-6, and NO	Ganeshpurkar and Saluja (2018)
		LPS-stimulated RAW 264,7 cells	*In vitro*	Reduced TNF-α, IL-1, and IL-6 levels	Suppression	TNF-α, IL-1, and IL-6 levels	Touzani et al. (2019)

### 5.1 *In vitro* immunomodulating effect of *Zingiber officinale*

An *in vitro* effect of several herbal products on transplant-related immune function in mixed lymphocyte culture demonstrated that ginger inhibited lymphocyte proliferation via the reduction of the IL-2 level (Wilasrusmee et al., 2002). A polyherbal formulation containing *Z. officinale* as one of the ingredients was shown to suppress IL-6 and TNF-α production in RAW 264.7 cells (Arora et al., 2020). On the contrary, another study reported that ginger exhibited an immunostimulant effect. The immunostimulant effect of dried ginger samples on RAW 264.7 murine macrophages and primary bone marrow-derived macrophages varied significantly depending on their drying conditions (Lee et al., 2019). Ginger processed at 50°C for 1 h exhibited the strongest macrophage activation by increasing the levels of IL-6 and TNF-α and IL-6, whereas samples processed by freeze-drying at −70°C and −90°C demonstrated little effect. The main underlying molecular mechanism for this immunosuppressive effect was suggested to be due to the induction of JNK, ERK, and p38 signaling pathways. The results suggested that drying conditions might cause chemical degradation, such as dehydration of gingerols to form shogaols. Drying could be used in the processing of ginger as a functional food with improved immunomodulating activity (Lee et al., 2019).

### 5.2 *In vivo* immunomodulating effect of *Zingiber officinale*

Jafarzadeh et al. (2014a) reported that experimental autoimmune encephalomyelitis (EAE) mice treated with ginger extract could reduce the expression of IL-33 and IL-27. The ginger extract, at doses of 200 and 300 mg/kg, enhanced the expression of IL-27 P28 and IL-27 EBI3. In contrast, it reduced the expression of IL-33 compared to peripheral blood smear (PBS)-treated EAE mice. *Z. officinale* extract modulated the expression of IL-33 and IL-27 in the spinal cords of EAE mice, thus ameliorating the clinical symptoms of the disease. Jafarzadeh et al. (2015a) reported in another study that IL-23 and IL-17 serum levels and expression of mRNA of IL-23 P19, IL-23 P40, and IL-17 in CNS were significantly reduced in EAE mice treated with 200 mg/kg ginger compared to control. However, in EAE mice treated with 300 mg/kg ginger, there was a significant decrease in serum levels of IL-17 and IL-23 and levels of P19, P40, and IL-17 expression in CNS. The findings suggested that ginger has the potential to be a source of lead molecules for development into a therapeutic agent to treat multiple sclerosis.

Ginger was shown to significantly decrease proinflammatory mediator levels, including TXB2, NO, IL-1β, and PGE2, in LPS-activated macrophages of the experimental mouse model of intermittent cold stress-induced fibromyalgia syndrome (FMS). The results indicated that the inflammatory state generated in the FMS model could be reduced by ginger (Montserrat-de et al., 2018). It was shown that the ethanol extracts of turmeric and ginger in a 1:1 ratio given to monosodium iodoacetate-induced osteoarthritis rats reduced the IL-1β level. The ethanol extract at 200 and 400 mg/kg could strongly decrease IL-1β, cartilage oligomeric matrix protein (COMP), hyaluronic acid (HA), malondialdehyde (MDA), and myeloperoxidase (MPO) serum levels compared to the control group not provided with the extract (Aborehab et al., 2017). The ginger extract could prevent OVA-stimulated allergic airway inflammation in Th2-mediated pulmonary inflammation in the mouse model, followed by *in vivo* inhibition of Th2-cell-driven response to the allergen*.* Eotaxin, IL-4, and IL-5 serum levels and specific IgE titers in mice were reduced after treatment with ginger compared to those of controls. The ability of ginger to decrease Th2-mediated immune responses indicated that it might have the potential for therapeutic use in allergic asthma (Ahui et al., 2008). Oral intake of a 2% ginger diet to mice with OVA-induced allergic rhinitis could reduce the secretion of IgE in serum, nasal rubbing, and severity of sneezing, reducing mast cell infiltration in nasal mucosa (Kawamoto et al., 2016).

Meanwhile, some studies demonstrated the ability of *Z. officinale* to protect our body from pathogens through stimulation of the immune system. The administration of the ginger aqueous extract at various doses (125, 250, and 500 mg/kg) in cyclophosphamide (CPA)-induced immunosuppress mice was able to inhibit CPA-induced immunosuppress changes compared with CPA control. At 250 mg/kg, the extract caused a decrease in body weight and gain and the weight of the thymus and spleen due to CPA treatment (Koo et al., 2015). The result agreed with a previous study that demonstrated that administration of ginger essential oil (100, 200, and 400 mg/kg) to sheep red blood cell-immunized mice recovered the immune response in CPA-induced immunosuppressed mice compared with the control (Carrasco et al., 2009). The immunostimulant effect of powdered ginger rhizome was also observed in fish rainbow trout (*Oncorhynchus mykiss*). Administration of a diet containing 1% powdered *Z. officinale* once a day for 12 weeks caused a significant immunostimulatory effect, exhibiting an increase in white blood cell, hematocrit, red blood cell values, respiratory burst, and lysozyme activities compared with the control group. The findings suggested that the dietary powdered ginger rhizome could ameliorate the immune system in rainbow trout (Haghighi and Rohani, 2013).

Qian et al. (2023) demonstrated that a ginger polysaccharide, UGP1; reduced tumor proliferation in tumor-bearing mice; raised the p53 expression and the ratio of Bax/Bcl-2; enhanced proinflammatory cytokine secretion, TNF-α, IL-2, IL-6; and decreased the secretion of pro-tumor cytokines TGF-β and bFGF in serum. The ethanol and aqueous extracts of *Z. officinale* decreased the inflammatory cell infiltration around the airways and decreased the elevated levels of IL-4 and IL-5 in the lungs and BALF of BALB/c mice compared to methylprednisolone as a control group (Khan et al., 2015; Harun and Mohamad, 2022). In another *in vivo* study, 6-gingerol in phosphate-buffered saline reduced IL-17 infiltration from the peripheral blood into the central nervous system, as well as neuroinflammation and demyelination (Han et al., 2019; Harun and Mohamad, 2022). In Albino rats with necrotizing enterocolitis, a whole ginger aqueous extract (1,000 mg/kg/day) lowered IL-1, TNF-α, and IL-6 levels (Cakir et al., 2018; Harun and Mohamad, 2022). The ethanol extract of *Z. officinale* at 450 mg/kg significantly reduced inflammatory cytokines (IL-6 and TNF-α), TGF-β1, anti-inflammatory cytokine (IL-10), angiogenesis (VEGF), and oxidative (MDA) factors, while increasing antioxidant factor glutathione (GSH) in peritoneal adhesions model of male Wistar rats (Yahyazadeh et al., 2023).

### 5.3 Clinical studies of *Zingiber officinale* on the immune system

Ginger administration to 120 participants in a randomized, double-blind, placebo-controlled clinical trial resulted in a decreased circulation of TNF-α and IL-1β in knee osteoarthritis patients. A capsule of ginger powder (500 mg) given once daily for 3 months to knee osteoarthritis patients showed decreased cytokine production. At baseline, IL-1β and TNF-α serum levels did not differ among the groups. However, the levels of both cytokines were reduced in the ginger group relative to the placebo group (500 mg starch) at 3 months. The findings suggested that taking a ginger supplement might have a beneficial effect on knee osteoarthritis treatment (Mozaffari-khosravi et al., 2016).

A preliminary clinical study was carried out to determine the immunomodulatory effects and pharmacokinetics of soft gel capsules containing *Echinacea angustifolia* (5 mg) and *Z. officinale* (25 mg) in 10 healthy volunteers (Dall'Acqua et al., 2019). Gene expression profiling displayed that the 542 distinct transcripts have variable levels of expression in PBMCs. These transcripts were separated into two transcriptional modules, one with 249 downregulated genes and the other with 293 upregulated genes. Interestingly, a bioinformatics study revealed that DEFA1, DEFA1B, and DEFA3 were among the most downregulated genes in PBMCs obtained from the subjects. An integrated examination of the gene expression data revealed that the anti-inflammatory and immunomodulatory effects of the formulation were comparable to those of hydrocortisone. *Z. officinale* at 300 mg daily was found to be beneficial in lowering various serum markers of the inflammation process, such as procalcitonin and eutrophillymphocyte ratio in individuals with community-acquired pneumonia (Reviono et al., 2023).

Numerous *in vitro*, *in vivo*, and clinical studies have been conducted to determine the immunomodulatory effects of *Z. officinale*. Mostly, the immunomodulatory effects of *Z. officinale* were investigated using macrophages and lymphocytes. Therefore, there is a necessity to elaborate on the immunomodulatory effects of the plant using other immune cells, such as monocytes, neutrophils, dendritic cells, and NK cells. More various animal disease models of immune disorders should be used to investigate the immunomodulatory effect of plant samples. *Z. officinale* samples were mostly tested in the form of crude aqueous and alcoholic extracts. The chemical composition of these extracts should be analyzed to determine their chemical marker for extract standardization. Extensive clinical studies, including phases 1, 2, and 3, should be performed before the extract can be developed into an immunomodulatory agent to treat various immune-related disorders in clinical services.

### 5.4 *In vitro* immunomodulating effects of *Zingiber zerumbet*

An *in vitro* study on the phagocytic activity of essential oil, zerumbone, and 80% ethanol extract of *Z. zerumbet* rhizomes on human leukocytes demonstrated that the oil possessed the strongest suppressive effect on chemotaxis activity. All samples inhibited CD18 integrin expression moderately and in a dose-dependent pattern. The strongest suppression of phagocytosis activity was displayed by the extract, with 55.43% of phagocytizing cells. Zerumbone significantly inhibited the oxidative burst of PMA- and zymosan-induced neutrophils. The presence of bioactive constituents, especially zerumbone, in the extract and oil might cause substantial inhibition by this extract and oil on the phagocytosis of neutrophils (Akhtar et al., 2019).

### 5.5 *In vivo* immunomodulating effects of *Zingiber zerumbet*

In an *in vivo* study, an 80% ethanol extract of *Z. zerumbet* at concentrations of 100, 200, and 400 mg/kg, when given for 15 days in male Wistar rats, led to strong immunosuppressive effects on the innate immune response (Ghazalee et al., 2019). The extract dose-dependently and significantly inhibited the chemotaxis of neutrophils, CD11b/CD18 complex expressions, phagocytic activity, and oxidative burst. Lysozyme and ceruloplasmin expressions in the rat plasma were also dose-dependently inhibited by the extract. Zerumbone was the main component of the extract as analyzed using chromatographic and spectroscopic methods. The findings indicated that the extract was a promising source for the development of an effective immunosuppressive drug due to its potent inhibitory effects on the innate immune system.

Chaung et al. (2008) reported that during short- and long-term treatment of the *Z. zerumbet* aqueous extract in an OVA-induced BALB/c mice model of anaphylaxis, there was inhibition of inflammatory mediator production and modulation of cytokine gene expression. The extract effectively suppressed the release of LTC_4_ from lung tissue *in vivo* and decreased the release of TNF-α and IL-4 *in vitro*. Treated animals also exhibited higher ratios of IFN-γ/IL-4 mRNA in their splenocytes compared to those of the control group. The ability of the *Z. zerumbet* extract to inhibit LTC_4_ synthesis and the modulation of Th1/Th2 cytokine production may indicate that it has therapeutic benefits in treating asthmatic patients.

I*n vitro* studies that evaluate the immunomodulatory effect of *Z. zerumbet* are very lacking. Extensive humoral and cellular response studies should be performed using various immune cells. Similar to *Z. officinale*, *in vivo* studies were conducted to investigate the effect of crude extracts of *Z. zerumbet* on the immune response using rats and mice. Zerumbone was found to be the main component as analyzed by chromatographic and spectroscopic methods. Clinical studies should be conducted to determine the effect of *Z. zerumbet* on the human immune response.

### 5.6 *In vitro* immunomodulating effects of *Zingiber cassumunar*

In an *in vitro* study, the ethyl acetate fraction of bangle rhizomes of *Z. cassumunar* at doses of 25, 50, and 100 μg/ml upregulated the IL-14 and IL-10 expression in mice macrophages (Nurkhasanah and Sofyan, 2019). The result agreed with a previous study that showed the immunostimulatory effect of several phenylbutenoid derivatives of *Z. cassumunar* rhizomes in mice macrophages. Among the compounds, (E)-4-(3′,4′-dimethoxyphenyl) but-3-en-1-ol has the highest immunostimulant activity (Chairul et al., 2008). Mahfudh et al. (2020a) demonstrated the *in vitro* effect of ethyl acetate extract (25, 50, and 100 μg/ml) of *Z. cassumunar* rhizomes on lymphocyte proliferation and phagocytic activity of mice macrophages. The extract at 100 μg/ml exhibited the strongest phagocytic activity as measured by phagocytosis index and an increase in active phagocytes. The extract also significantly increased lymphocyte proliferation compared to the negative control.

Mahfudh et al. (2020b) demonstrated in another study that the bangle extract of *Z. cassumunar* (25, 50, and 100 μg/ml) exhibited an immunomodulating effect on NO and ROI secretions, and levels of IL-14 and IL-10 expression in mice macrophages. The extracts at 25 and 50 μg/ml significantly increased ROI secretion and expression levels of IL-10 and IL-14, but the level of NO was decreased. The extract of *Z. cassumunar* and its hexane and chloroform fractions were evaluated for their phagocytic activity on mice macrophage cells. Among the samples, the chloroform fraction demonstrated the strongest phagocytic activity. Bioassay-guided isolation of the chloroform fraction yielded (E)-4-(3,4-dimethoxyphenyl) but-3-en-1-ol as the active compound (Ramadhan et al., 2020). The aforementioned findings indicated that the *Z. cassumunar* extract has the potential to be an important source for the development of an immunostimulant.

### 5.7 *In vivo* immunomodulating effects of *Zingiber cassumunar*

The immunomodulating effect of *Z. cassumunar* ethanol extract administered at 250, 500, and 1,000 mg/kg for 7 days in LPS-activated mice displayed that the extract was able to increase the ROI and NO secretion levels. However, the phagocytic activity of the macrophages was not increased by the extracts (Nurkhasanah and Fauziah, 2017). NO and ROI are involved in important functions in the activation of disease-fighting mechanisms. NO and ROI also interact in immune system activation, where macrophages kill incoming pathogens (Bogdan et al., 2000). Concomitant treatment with Phlai capsules (containing *Z. cassumunar* extract) decreased bronchial hyperresponsiveness and significantly improved symptom scores in adult asthmatic patients with partially controlled symptoms with ICS, especially in patients with bronchial hyperresponsiveness and high FeNO levels at baseline (Dulpinijthamma et al., 2023).

Some *in vitro* studies have been performed using various immune cells, such as macrophages and lymphocytes, to evaluate the immunomodulatory effects of *Z. cassumunar*. However, few *in vivo* studies have reported on the immune effect of the plant extract. Moreover, the metabolite profiles of *Z. cassumunar* were not determined. For clinical studies, sufficient preclinical testing should be performed using standardized extracts before they can be subjected to clinical studies.

## 6 Immunomodulating effects of *Alpinia* species

Many *Alpinia* species, especially *A. galanga*, *A. oxyphylla*, *A. zerumbet*, A. *purpurata*, *A. officinarum*, *A. katsumadai*, and *A. conchigera*, have been evaluated for their immunomodulating effects. The immunomodulatory effects of the *Alpinia* species are depicted in Table 1, whereas the immunomodulatory effects and mechanisms of some bioactive compounds of the *Alpinia* species are shown in Table 2.

### 6.1 *In vitro* and *in vivo* immunomodulating effects of *Alpinia galanga*

*In vitro* study on the anti-psoriatic effect of the *A. galanga* ethanol extract in HaCaT keratinocyte cells has indicated that the extract could modulate NF-κB signaling biomarkers expression to treat psoriasis. According to semi-quantitative RT-PCR analysis, the extract significantly enhanced TNF-α induced protein 3 expression and markedly inhibited the expression of mRNA of CD-40, NF-κB2, and CSF-1 (Saelee et al., 2011). George et al. (2021) reported that the *A. galanga* extract displayed inhibitory activity on the release of proinflammatory mediators (ROS, TNF-α, IL-6, and NO). It also enhanced the release of IL-10 in LPS-enhanced RAW 264.7 cells. The extract also showed inhibition on the release of inflammatory enzymes (COX-2, iNOS, and MMP-9) by inhibiting LPS-induced activation of JAK/STAT and TLR4 pathways, particularly JNK, p38, IκBα, and STAT phosphorylation.

The immunomodulation activity of the flavonoid fraction of *A. galanga* methanol extract was determined in mice by measuring delayed-type hypersensitivity reaction (DTHR), splenocyte proliferation, and T-cell proliferation. The extract at 100 mg/kg bw significantly enhanced the proliferation of splenocytes and T cells in the spleen. Chromatographic analysis of the flavonoid fraction indicated the presence of quercetin (Jain et al., 2012).

### 6.2 *In vitro* and *in vivo* immunomodulating effects of *Alpinia oxyphylla*

Lee et al. (2019) reported the anti-osteoarthritis and anti-inflammatory activities of the extract of *A. oxyphylla*. At a dose of 100 μg/ml, the extract displayed inhibition on the PGE2, NO, IL-6, TNF-α, and IL-1β expression and the activation of p38 MAPK, extracellular signal-regulated kinase (ERK), and JNK in LPS-activated RAW 264.7 cells. In an *in vivo* study to evaluate anti-osteoarthritis activity in an osteoarthritis rat model induced by monosodium iodoacetate, the extract at 150 or 300 mg/kg reduced proinflammatory cytokine and mediator release, prevented cartilage degradation, and attenuated joint pain. In other *in vivo* studies, the 50% ethanol extract of *A. oxyphylla* also exhibited nociceptive and anti-inflammatory activities. The *A. oxyphylla* extract significantly inhibited ear thickness, abdominal constriction, and paw edema compared with the control. Explants treated with the extract (50–400 μg/ml) suppressed proteoglycan degradation by IL-1α. *In vitro* assay showed that the extract suppressed LOX, COX-1, and COX-2. The outcomes of this study suggested that the *A. oxyphylla* extract might be beneficial in the treatment of osteoarthritis and associated symptoms (Yu et al., 2020).

When the *A. oxyphylla* fruit ethanol extract was orally administered to the experimental autoimmune encephalomyelitis (EAE) mouse model, it was able to reduce EAE symptoms. The extract reduced inflammation, axonal swelling, gliosis, and demyelination in the spinal cord. Immunohistochemistry analysis and quantitative PCR indicated that the extract-treated group also decreased CD11b+ monocytes, CD4^+^, and CD8^+^ T-cell infiltration. In the extract-treated mice spinal cords, IL-17, IFN-γ, Th1 transcription factor T-bet, and Th17 transcription factor retinoic acid receptor-related orphan receptor γ (RORγt) were downregulated. In an *in vitro* study, yakuchinone A isolated from the extract could reduce the production of IL-17 and inhibit the EAE symptoms. The outcome of this study revealed that the extract alleviated the symptoms of EAE in mice, which might be associated with the regulation of Th1/Th17 response. *A. oxyphylla* might have the potential to be developed into a therapeutic agent to treat multiple sclerosis (Huang et al., 2019). After 14 days of treatment with *A. oxyphylla* fructus extract (360 mg/kg), the LPS-induced Alzheimer’s disease model demonstrated varying degrees of improvement as determined by the Y-maze test, the Morris water maze test, and histopathological investigation. Furthermore, ELISA results indicated that petroleum ether extracts decreased the elevated levels of IBA-1, IL-1β, Aβ_1-42_, and p-tau in the hippocampus and cortex following LPS treatment (Wang et al., 2018; Guo et al., 2022).

### 6.3 *In vitro* and *in vivo* immunomodulating effects of *Alpinia zerumbet*

Among the methanol extracts of the leaves, placenta, pericarps, and seeds of *A. zerumbet*, the placenta extract showed the strongest NO inhibitory activity. Kavalactone derivatives were identified as the major compounds contributing to the anti-inflammatory activity of the extracts (Nishidono et al., 2020). The *A. zerumbet* extract reportedly exhibited antihypertensive effects by enhancing 3T3-L1 intracellular cAMP and accelerating the vasorelaxant response, which has potential anti-obesity effects in addition to anti-diabetic complication and hypolipidemic effects (Xiao et al., 2020). It could reduce oxidative stress in the NOS-NO signaling pathway and exhibited anti-inflammatory and antioxidant effects on the NF-κB signaling pathway. Another study observed that *A. zerumbet* fruit essential oil was shown to suppress NF-κB signaling in human umbilical vein endothelial cells (HUVECs) treated with high levels of glucose. Pre-treatment of HUVECs with the oil (0.25, 1.0, and 4.0 μg/ml) inhibited the levels of IL-8, TNF-α, ICAM-1, and VCAM dose-dependently. The oil also suppressed the translocation of the p65 subunit of NF-κB to the endothelial cell nucleus in a dose-dependent pattern (Huang et al., 2017).

### 6.4 *In vivo* immunomodulating effects of *Alpinia officinarum*

Ni et al. (2022) evaluated the regulating effect of a water-soluble polysaccharide (AOHP) isolated from *A. officinarum* on mouse immunity after oral administration. AOHP was made up of T-α-D-Glcp, (1,4)-α-D-Glcp, and (1,4,6)-α-D-Glcp with a number-average molecular weight of 26.0 kDa. Treatment with AOHP improved the innate immune status of the mice, and no obvious toxicity was observed. In another study, amine surface-functionalized mesoporous silica nanoparticles (MSN) of *A. officinarum* extract were shown to increase the immune status of rainbow trout (*Oncorhynchus mykiss*). All treatment groups demonstrated improvements in immunological markers. However, the fish group given 1.5% extract + MSN had the greatest significant differences in total protein, myeloperoxidase content, lysozyme content, and antiprotease activity. Most immune-related genes were significantly upregulated in the group that received 0.5% extract + MSN. The findings suggested that the encapsulated *A. officinarum* can be used in fish diets as a supplement to stimulate the immune response and increase its resistance to infectious diseases (Awad et al., 2020).

### 6.5 *In vitro* immunomodulating effects of *Alpinia katsumadai*

The *in vitro* anti-asthmatic and antioxidant effects of *A. katsumadai* seed ethanol extract on a murine OVA-stimulated asthma model were examined by observing the levels of Th2-type cytokines, lung microscopic examination, and eosinophil recruitment. The extract effectively suppressed IgG2a, IgE, eosinophilia, and mucus hypersecretion and significantly inhibited the increase in mRNA expression and Th2-type cytokines, including IL-5 and IL-4 in lung tissue and bronchoalveolar lavage fluid (BALF) in the asthmatic mouse model. Treatment with the *A. katsumadai* extract also reduced the generation of ROS in BALF. The results indicated that *A. katsumadai* can be used to treat asthma (Lee et al., 2010).

The investigations on the immunomodulatory effects of *Alpinia* species were mostly based on the non-standardized plant extracts, whereas the bioactive constituents of the plants contributing to the immunomodulating activities were mostly not identified. Extensive *in vitro* and *in vivo* studies should be performed to determine the immunomodulatory effects of *Alpinia* species. Similar to *Zingiber* species, the crude extracts of plant samples were used in animal models to evaluate their *in vivo* immunomodulating effect. Pharmacokinetic and toxicological studies using standardized extract should be performed before they can be subjected to clinical studies.

## 7 Immunomodulating effects and mechanisms of bioactive metabolites

### 7.1 Gingerols and shogaols

Several studies have reported that gingerols and shogaols alleviated inflammatory mediators and proinflammatory cytokine generation and the release of histamine. Van Breemen et al. (2011) demonstrated that 10-gingerol, 10-shogaol, and 8-shogaol isolated from *Z. officinale* could inhibit inflammatory mediators of COX-2 with IC_50_ values of 32, 7.5, and 17.5 μM, respectively, but there is no inhibitory activity reported in COX-1. It has been demonstrated that 6-shogaol reduced mast cell-activated allergic responses by inhibiting histamine and proinflammatory cytokines release. This is accomplished via the regulation of NF-κB and phosphorylation of JNK. 6-Shogaol at 1 and 5 mg/kg could decrease the passive cutaneous anaphylaxis reaction by 44.9% and 72.1%, respectively. Furthermore, 6-shogaol at 0.1, 1, 10, 50, and 100 mM markedly suppressed histamine release activated by secondary metabolite 48/80 in rat peritoneal mast cells. At 100 mM, the histamine content of rat peritoneal mast cells was reduced by approximately 51%. At 0.1, 1, 10, 50, and 100 mM, 6-shogaol suppressed the production of IL-6 and TNF-α in human mast cells (HMC-1). Treatment with 6-shogaol also lowered IL-8 secretion by a significant amount. Pre-treatment of HMC-1 cells with 50 and 100 mM of 6-shogaol caused significant inhibition on the activation of NF-kB p65 elicited by PMA plus A23187 via stabilizing IkB-α. In addition, after treatment with 6-shogaol, JNK activation in response to PMA plus A23187-induced HMC-1 cells was markedly reduced (Sohn et al., 2013).

Furthermore, 6-, 8-, and 10-gingerols administered at a dose of 0.15 μmol/L each to human T cells in an *in vitro* model for 48 h demonstrated an enhancement in inflammatory cytokine levels compared to T cells that were not given gingerols intervention. However, the rate at which these cytokines increased was not reported. In addition, the administration of 8-gingerol at concentrations of 0.03–2.7 μmol/L increased IFN-γ levels at 20%–30%. The administration of 10-gingerol at concentrations of 0.03–1.2 μmol/L increased IFN-γ levels by 15% (Schoenknecht et al., 2016). At 20 μM, 10-gingerol, 6-shogaol, 8-shogaol, and 10-shogaol reduced the secretion of IL-1β on LPS-primed and ATP-activated IL-1β secretion in THP-1 macrophages with the inhibitory values of 102.2, 95.2, 63.6, and 46.5%, respectively (Ho & Chang, 2018).

6-Gingerol significantly increased the IL-10 level and decreased the Il-17 level in both bowel tissues and serum in dextran sulfate sodium (DSS)-stimulated ulcerative colitis mice model. Furthermore, 6-gingerol might reduce IκBα and p65 phosphorylation levels, which were increased by DSS, indicating that it might reduce inflammation by regulating the NF-κB signaling pathway (Sheng et al., 2020). Furthermore, it was reported that 6-gingerol inhibited the production of cytokines of Th1 and Th2 in OVA-activated BALB/C mouse spleen cells. 6-Gingerol suppressed the differentiation and expansion of Th1 and Th2 cells from T lymphocytes resulting in the reduction and prevention of allergic rhinitis symptoms (Kawamoto et al., 2016). These results indicated that gingerols and shogaols have immunomodulatory activity by decreasing the production of COX-2 and proinflammatory cytokines and the release of histamine, and they reduced the severity of inflammatory disorders.

The anti-neuroinflammation effect of the *Z. officinale* extract and its seven gingerol-related secondary metabolites was investigated on LPS-activated BV2 microglia. It was found that gingerols and shogaols at 20 μM showed inhibition on the IL-6, TNF-α, IL-1β, and NO release and their mRNA levels, except for zingerone and 6-gingerol. The inhibition of the proinflammatory gene expression was due to the blocking of NF-κB activation. The anti-neuroinflammatory properties of gingerols were improved by lengthening the alkyl chain but conversely attenuated those of shogaols. It was suggested that the strong anti-neuroinflammatory activity of the fresh ginger extract was largely contributed by 10-gingerol (Ho et al., 2013).

In addition, 6-, 8- and 10-gingerols/shogaols were shown to inhibit the canonical (NOD)-like receptor family pyrin domain-containing 3 (NLRP3) inflammasome-mediated IL-1β secretion in ATP- and LPS-stimulated THP-1 macrophages. After treatment with 20 M of 10-gingerol and all of the shogaols, the canonical IL-1 secretion was significantly decreased. It was found that shogaols exhibited a stronger inhibitory effect than the corresponding gingerols, and 6-shogaol was the most potent. However, extended alkyl chains have a negative effect on the inhibitory effect of shogaols. The results revealed that gingerols and shogaols might have the potential to be developed as NLRP3 inflammasome-mediated IL-1β secretion inhibitors for the prevention of NLRP3 inflammasome-associated diseases (Ho and Chang, 2018).

### 7.2 Zerumbone

Zerumbone is the major bioactive constituent of *Z. zerumbet*. Zerumbone at concentrations of 3.13, 12.5, and 50 μg/ml depicted a decrease in the CD18 expression in polymorphonuclear neutrophils stimulated by LPS *in vitro* in a dose-dependent way. The expression of CD18 when administered with 50 μg/ml was 72.57%, which was lower than the control group (92.47%). A decrease in the CD18 expression led to a decrease in the inflammatory process, indicating that zerumbone has the potential to be developed into an immunosuppressant (Akhtar et al., 2019). Another study revealed that zerumbone (25, 50, and 100 mg/kg) suppressed the phagocytic activity in a concentration-dependent manner compared to the control group. Zerumbone also showed a significant and concentration-dependent way of decreasing T and B lymphocyte proliferation, as well as releasing Th1 and Th2 cytokines. Zerumbone at 100 mg/kg also significantly inhibited the expression percentage of CD8^+^ and CD4^+^ in splenocytes (Jantan et al., 2019).

Zerumbone showed significant inhibition on the upregulation of proinflammatory mediators, such as TNF-α, COX-2, PGE2, and IL-1β in LPS-activated human macrophages. It also substantially decreased IKKα/β, IκBα, and NF-κB (p65) phosphorylation and restored IκBα degradation. In line with it, zerumbone also dose-dependently exhibited an extraordinary reduction of the p38 MAPKs, ERK, Akt, and JNK expression. It also decreased the signaling expression of MyD88 and TLR4, involved in the PI3K-Akt, MAPK, and NF-κB activation process. The relative gene expression of COX-2, IL-1β, and TNF-α was significantly reduced at a higher dose of 50 μM. The elevation of the transcription rate of mRNA from the proinflammatory mediator-induced processes expressed in LPS-stimulated U937 macrophages was also significantly decreased by zerumbone. It is concluded that zerumbone has strong inhibition on inflammatory markers activation in the signaling pathways (Haque et al., 2018). In agreement with Mohamad et al. (2015), zerumbone alone and in nanostructured lipid carrier (NLC) encapsulated suppressed levels of IL-6 and IL-1β and upregulated the levels of serum IL-2 and IFN-γ. NLC zerumbone increased the cytotoxic effect on natural killer T cells, T cells, and helper T cells in spleens. The results suggested that zerumbone has the potential to be developed into a potent therapeutic and preventive agent for the management of various inflammatory and mediated immune disorders.

In a model of LPS- and IFN-γ-induced RAW 264.7 murine macrophages, zerumbone was reported to dramatically decrease iNOS synthase and COX-2 protein production (Murakami et al., 2002; Nuzzo et al., 2022). It inhibited methicillin-resistant *Staphylococcus aureus* growth by depolarizing the membrane, increasing membrane permeability, and lastly breaking the cell membrane and killing the germs. Zerumbone increased cell membrane permeability and decreased membrane potential, resulting in membrane structural integrity loss and bacterial death. Based on 2′,7′-dichlorofluorescein diacetate (DCFDA) dye analysis, zerumbone reduced ROS production by methicillin-resistant bacteria (Albaayit et al., 2022). Concomitant administration of zerumbone and curcumin revealed a considerable reduction of tumor size in colorectal cancer in BALB/c mice. This combination increased miR-34a in CRC cell lines and tumor tissue (Nobari et al., 2023). Zerumbone strongly inhibited the oxidative burst of zymosan and phorbol myristate 13-acetate (PMA)-stimulated neutrophils. It significantly decreased extracellular ROS generation in PMNs, with an IC_50_ value of 17.36 M, comparable to aspirin (Akhtar et al., 2019; Ibanez et al., 2023). Zerumbone was also effective against fluconazole-resistant and fluconazole-susceptible *Candida albicans* biofilms and disrupted the extracellular matrix (Abreu-Pereira et al., 2023).

### 7.3 1’-Acetoxychavicol acetate and hydroxychavicol acetate

1’-Acetoxychavicol acetate (ACA) and hydroxychavicol acetate are naturally occurring polyphenols that have been isolated from the rhizomes of *A. galanga*. They have potent anti-inflammatory and antioxidant activities. ACA exhibited anti-inflammatory, immunomodulatory, antioxidant, anti-obesity, anti-cancer, antiallergic, and anti-asthma effects (Ohnishi et al., 2012; Williams et al., 2013). AMP-activated protein kinase (AMPK), an enzyme that controls signal transduction pathways and is essential in the prevention of illnesses such as obesity, cancer, neurodegenerative disorders, hyperlipidemia, and diabetes, was activated in response to ACA. These findings indicated the vital role played by AMPK in many ACA pharmacological effects, which is effective in the prevention of serious illnesses (Yuasa and Yuasa, 2020). ACA and hydroxychavicol acetate (HCA) isolated from *A. galanga* rhizomes were evaluated for their effect on the production of cytokines in Th cells. High antioxidant action and enhanced cell apoptosis displayed by ACA resulted in reduced production of cytokines by Th cells. However, HCA did not show antioxidant and apoptotic activities. It attenuated the expression of IFN-γ and enhanced the production of IL-2 in the Th cells. HCA was also shown to suppress T-bet expression, which is involved in the induction of IFN-γ, suppression of IL-2 in TH cells, and suppression of T-bet-stimulated Th1 cell differentiation. HCA has the potential to be used to treat immune-inflammatory diseases due to excessive Th1-activated immune responses (Min et al., 2009).

In OVA-induced asthma animal models, the ACA-treated group dose-dependently exhibited inhibition of eosinophil infiltration and a reduction in IgE level. Lung histopathology indicated that ACA given at 50 mg/kg/day could reverse the OVA-induced alterations such as eosinophil infiltration, mucus plugs, goblet-cell hyperplasia, and airway remodeling. In addition, ACA decreased the expression of T-helper type 2 cytokines and inhibited T-helper type-1 cytokine release, overexpressed during asthma induction. The antiasthmatic effect of ACA was suggested by its ability to work on immunological and inflammatory pathways (Seo et al., 2013). Moreover, ACA also inhibited NF-κB activation and invasiveness in cellular models (Aggarwal and Ichikawa, 2005). It showed a strong inhibitory effect against the release of IL-4, TNF-α, and β-hexosaminidase from RBL-2H3 cells after a challenge with an antigen (Matsuda et al., 2004).

ACA isolated from *A. galanga* inhibited the infiltration of leukocytes, especially eosinophils, in a mouse model of OVA-induced asthma. ACA, given at doses of 25 and 50 mg/kg/day to the animal model, suppressed the IgE level and the infiltration of eosinophils in the lungs. Histopathological alterations, such as glycoprotein secretion, goblet-cell hyperplasia, eosinophil infiltration, and airway remodeling, were inhibited. Additionally, ACA effectively suppressed cytotoxic T cells of CD4^+^ and CD8^+^ Th cells. CD8^+^ cytotoxic T cells and CD4^+^ Th cells are important in triggering asthma. Furthermore, ACA suppressed the expression of Th2 cytokines (IL-4, IL-6, and IL-13) and Th1 cytokines (IL-12α and IFN-γ) in the ACA-treated group in a dose-dependent manner. These findings suggested that ACA has great potential to be developed as an antiasthmatic agent (Seo et al., 2013).

### 7.4 Cardamomin

Cardamomin (2′,4′-dihydroxy-6′-methoxychalcone) is a natural chalcone with strong anti-inflammatory activity. It is found in some *Alpinia* genera, such as the seeds of *A. katsumadai*. Cardamomin from *A. katsumadai* was found to possess an anti-inflammatory effect by its ability to decrease paw edema after carrageenan administration. The decrease in paw edema by cardamomin was suggested to be related to the reduction in the levels of COX-2, NO, TNF-α, MDA, IL-1β, IL-6, and iNOS in the paw edema. The secondary metabolite also blocked the degradation of NF-κB and IκBα and the phosphorylation of MAPKs (p38, JNK, and ERK). Cardamomin also upregulated the expression of heme oxygenase-1 (HO-1) in paw edema (Li et al., 2015).

The effects of cardamomin from *A. conchigera* on LPS-induced mortality and activation of NF-κB in LPS-induced RAW 264.7 cells have been investigated. The secondary metabolite dose-dependently reduced the NF-κB reporter gene expression that was stimulated by TNF-α or LPS. Cardamomin effectively suppressed the LPS-activated generation of TNF-α and NO and the expression of COX-2 and iNOS in RAW 264.7 cells. Cardamomin also blocked the activation of inhibitor κBα (IκBα), nuclear translocation of NF-κB, and both the degradation and phosphorylation of inhibitor κBα (IκBα). Cardamomin also suppressed the LPS-activated activation of Akt but did not suppress IκB kinases directly. In addition, the secondary metabolite downregulated Ser536 phosphorylation of the RelA/p65 subunit of NF-κB and transcriptional activity. It also significantly suppressed the activation of p38 MAPK. It was also reported that C57BL/6 mice pre-treated with cardamomin could rescue them from LPS-activated mortality in conjunction with the decreased TNF-α serum level. The findings suggested that cardamomin has the potential to be developed into a drug candidate for the suppression of NF-κB-associated pathological inflammatory conditions (Lee et al., 2006).

The anti-arthritic effect of cardamomin has been evaluated in a rheumatoid arthritis (RA)-induced rat model, specifically on the pain and inflammatory response of RA. RA paw inflammation in the rat was induced by Freund’s adjuvant (CFA). Cardamomin at doses of 0.625, 1.25, 2.5, and 5.0 mg/kg significantly inhibited RA-activated pain, inflammatory responses, and the progression of joint deterioration in rats. The plasma levels of IL-1β, IL-6, and TNF-α in rats treated with cardamomin were markedly inhibited. The outcomes of the study revealed that cardamomin possessed strong anti-arthritic properties in a CFA-induced RA rat model (Voon et al., 2017).

Park et al., 2014 reported that cardamomin has an anti-nociceptive effect as it dose-dependently inhibited Tgase-2, p65, and COX-2 and restored IκB expression in MG63 osteoblast-like and RAW 264.7 cells. Cardamomin suppressed the NF-κB activation and target genes of NF-κB (COX-2, TNF-α, and iNOS) in LPS-induced RAW 264.7 cells. Cardamomin-treated cells suppressed the degradation and resynthesis of IκBα protein. Furthermore, cardamomin suppressed the phosphorylation of Akt, a protein that regulates the NF-κB transcription through a mechanism dependent on RelA/p65 phosphorylation. Obviously, cardamomin dose-dependently inhibited the transcription activity of the RelA/p65 domain and significantly inhibited the RelA/p65 phosphorylation at Ser536, an essential step for NF-κB transcriptional activation. Among MAPKs (JNK/SAPK, p38, ERK1/2, and p38), which are important for NF-κB activation, cardamomin induced the phosphorylation of p38 but did not inhibit the LPS-stimulated activation of JNK/SAPK and ERK1/2 (Lee et al., 2006).

### 7.5 Yakuchinones

Yakuchinone A (l-[4′-hydroxy-3′-methoxyphenyl]-7-phenyl-3-heptanone) and yakuchinone В (l-[4′-hydroxy-3′-methoxyphenyl]-7-phenylhept-l-en-3-one) are diarylheptanoids of *Alpinia oxyphylla*. *In vitro* studies have shown that both secondary metabolites exhibited strong inhibitory effects on leukotrienes and prostaglandins syntheses. The yakuchinones inhibited the iNOS and COX-2 expression as well as TNF-α mRNA expression in mouse skin treated with 12-O-tetradecanoylphorbol-13-acetate (TPA). Both secondary metabolites also suppressed the TPA-activated DNA binding activity of NF-κB when applied topically on mouse skin (Chun et al., 2002). The anti-inflammatory effect of yakuchinone A and its ability to improve psoriasis dermatitis have been determined by Kim and Hwang (2020). Yakuchinone A attenuated the mRNA expression of IL-6, IL-8, and TNF-α in the presence of TNF-α on human epidermal keratinocytes (HaCaT cells). Yakuchinone A also suppressed a chemokine, CC motif ligand 20 (CCL20), that drew immune cells like Th17 cells to the inflammation sites. Yakuchinone A dose-dependently suppressed STAT3 and IκB and the phosphorylation involved in the CCL20 expression. The results indicated that yakuchinone A has the potential for development into a new therapeutic agent to treat psoriasis dermatitis.

### 7.6 1,8-Cineole

1,8-Cineole (1,3,3-trimethyl-2-oxabicyclo[2.2.2]octane) is a bicyclic monoterpene found in the essential oils of many plants of the Zingiberacea family, including *Zingiber* and *Alpinia* species (Brown et al., 2017; Xiao et al., 2020). The anti-inflammatory and analgesic properties of 1,8-cineole have been well documented (Jain et al., 2012). In LPS-induced alveolar macrophages, 1,8-cineole significantly reduced the levels of inflammatory mediators (IL-1α, IL-1β, TNF-α, and NO) (Yadav and Chandra, 2017). In human unselected lymphocytes and LPS-activated monocytes, 1,8-cineole inhibited the production of cytokines such as IL-4, IL-5, IL-1β, and TNF-α in lymphocytes. Moreover, cytokine monocyte production of IL-6, IL-8, TNF-α, and IL-1β was also inhibited. The outcomes of these studies indicated that 1,8-cineole was a strong inhibitor of TNF-α and IL-1β (Juergens et al., 2004).

In OVA-sensitized guinea pigs, 1,8-cineole was administrated by inhalation and showed an anti-inflammatory effect. TNF-α and IL-1β in BALF were lower in the 1,8-cineole-treated group than in the untreated group. Moreover, 1,8-cineole reduced mucociliary clearance stimulated by the antigen presentation (Bastos et al., 2011). In an acute lung injury of the mouse model, 1,8-cineole at 30 mg/kg also reduced IL-1β and TNF-α levels and enhanced the IL-10 level in LPS-stimulated lung tissues. IL-1β and TNF-α levels in lung homogenate were significantly enhanced 6 h after the LPS challenge. Furthermore, 1,8-cineole reduced the NF-κB expression and TLR4 and reduced neutrophils and macrophages in BALF (Zhao et al., 2013). 1,8-Cineole also has a potential beneficial effect in acute pancreatitis. In acute pancreatitis-induced mice, 1,8-cineole ameliorated cerulein-stimulated pancreatic edema, histological damage, and expression of NF-κB. 1,8-Cineole pre-treatment at 100, 200, and 400 mg/kg orally inhibited the upregulation of IL-6, TNF-α, and IL-1β levels due to cerulein induction, but IL-10 level was increased by 1,8-cineole (Lima et al., 2013). Ibuprofen and 1,8-cineole (15 and 10 mg/kg, respectively) enhanced cognitive performance and reduced anxiety in a rat model of oxidative stress hyperammonemia. This combination also reduced the expression of the genes IL-6 and IL-1β (Bahrami et al., 2023). T-cell subset distribution and PD-1 and PD-L1 expression were examined in chronic rhinosinusitis patients with monocyte subsets (CRSwNP and CRSsNP). Chronic rhinosinusitis caused a shift in the inflammatory phenotypes of peripheral monocyte subsets, which may be reversed by 1,8-cineol (Polasky et al., 2021). 1,8-Cineol concentrations in tissue samples of nasal polyps from CRSwNP patients were significantly higher than those in the control group (MacKenzie et al., 2023).

### 7.7 Lectin

Lectin, a carbohydrate-binding protein, can be found in several plants, including *Alpinia purpurata* (Brito et al., 2017). The lectin found in *A. purpurata* has modulatory effects on immune cells by inducing cell division, migration, proliferation, and activation of cell death. The effect was due to the binding of the glycan moieties on immune cell surfaces. This binding initiated biological responses, inhibition, or amplification of transmembrane signal transduction pathways (Geijtenbeek et al., 2016). Furthermore, the interaction of lectin and immune cells might trigger cytokine production. Lectin affected PBMCs to release IL-17A, TNF-α, IL-10, and IL-6. Moreover, lectin stimulated the production of IFN-γ and NO (Brito et al., 2017). IFN-γ, produced by NK cells and T CD4^+^ lymphocytes, play a crucial role in macrophage activation, differentiation, and induction of proinflammatory expression such as iNOS (Duque et al., 2014; Sol et al., 2021). Lectin enhanced the production of the Th1/Th17 phenotype and IFN-γ, IL-6, TNF-α, and IL-17 (Brito et al., 2017). Lectin was reported to be able to stimulate innate immune cells and T-helper cells (Kankia, 2015). In an experimental murine model of schistosomiasis, cytokine production in PBMC supernatants of infected patients showed an elevation of IL-5 production, which was higher than that induced by soluble egg antigens alone (Reis et al., 2008). Lectin at 5.0 µg increased globulin and creatine kinase MB (CK-MB) isoenzyme, increased the neutrophils expression in the heart and lung, and decreased IL-10 levels (Brito et al., 2017). In an *in vitro* study, lectin decreased the production of NO from mouse peritoneal macrophages and inhibited mononuclear cell proliferation after being stimulated by mitogen Con A (Ditamo et al., 2016). Seervi and Ahmed (2018) reported that lectin stimulated PBMC proliferation. Moreover, 3 h of lectin treatment showed increased mRNA levels of IL-1β, IFN-γ, and IL-6, whereas TGF-β increased in 6 h treatment.

### 7.8 Rutin

Rutin, commonly known as vitamin P, is a flavonoid found widely dispersed in the Zingiberaceae family, including the leaves of *A. zerumbet* and *A. purpurata* (Victório et al., 2010a). Rutin has been investigated for various pharmacological effects, such as immunomodulation, antioxidant, antibacterial, and anti-inflammatory (Zhao et al., 2020). An *in silico* analysis of rutin indicated that it might dock with the active sites of NO, IL-1, IL-6, and TNF-α and revealed positive interactions with these targets. The hydrogen bonding with chain A at Gly-121 on the backbone of TNF-α allowed for docking, and the sugar molecule also demonstrated hydrogen bonding with chain A at Tyr-59. The flavonol nucleus displayed hydrogen bonding to Arg-4, Phe-46, Gln-48, and Lys-103 upon interaction with IL-1. Furthermore, NO interaction with hydrogen bonds to Ser-339, Trp-683, Val-65, Asp-601, and Hem-801 was demonstrated by rutin and IL-6, as well as by helix A and helix D. Rutin interacted with chemokines and inflammatory mediators (Ganeshpurkar and Saluja, 2018). Rutin dramatically increased IFN-γ production during lymphocyte activation following stimulation by mitogens or antigens, but it had little effect on PBMC proliferation (Cherng et al., 2008). Through cellular and humoral pathways, rutin displayed immunostimulating action. In the hemagglutination test, rutin significantly increased immunoglobulin levels, DTHR, and antibody titer, brought on by sheep red blood cells. Additionally, rutin improved the phagocytic index in the carbon clearance assay and restored leukocyte function in cyclophosphamide-treated rats (Ganeshpurkar and Saluja, 2018).

Furthermore, Luo et al. (2020) discovered that rutin, given orally at doses of 25, 50, and 100 mg/kg, dramatically raised antibody titer and immunoglobulin levels and ameliorated DTHR. Also, it could greatly restore leukocyte and phagocytosis function, revealing that rutin has the potential to play an important role in boosting immunological activity through cellular and humoral-mediated processes. This study showed that rutin might improve thymus index, decrease the rise in spleen index brought on by immunological stress, preserve the integrity of spleen bodies and thymus lobules, and prevent inflammatory mediator exudation, all of which could have a protective effect on immune organs (Lan et al., 2022). In addition to the reduction of NO, IL-6, and ROS generation in RAW 264.7 cells, rutin also acted as an immunological regulator by reducing the levels of the mRNA expression of iNOS, NF-κB, and COX-2 (Alhoshani et al., 2017). Rutin was found to have a greater inhibitory effect on IL-1, IL-6, and TNF-α levels in LPS-activated RAW 264.7 cells at different doses ranging from 5 to 100 M (Touzani et al., 2019). It exhibited its immunomodulatory effect by decreasing the expression levels of MyD88, TLR4, TRAF6, and p65 genes and increasing the expression levels of I-B gene, MyD88, TLR4, TRAF6, phosphorylation p65, and phosphorylation I-B proteins in TLR4- NF-κB signaling pathway. The expression levels of the non-phosphorylated IB and p65 proteins, however, were unaffected. In other words, rutin could activate the TLR4-NF-B signaling pathway, which has an immunomodulatory regulatory impact (Lan et al., 2022).

### 7.9 Other bioactive secondary metabolites

A diarylheptanoid, 7-(4′-hydroxy-3′-methoxyphenyl)-1-phenylhept-4-en-3-one (HMP) isolated from *A. officinarum* was shown to possess an anti-inflammatory effect in LPS-activated mouse RAW 264.7 macrophages and human PBMCs *in vitro* (Yadav et al., 2003) HMP at the concentration range of 6.25–25 μM significantly reduced the NO level in the mice macrophages and IL-1β and TNF-α levels in PBMCs in a dose-dependent pattern. Additionally, HMP suppressed iNOS, COX-2 protein, and mRNA expression in RAW 264.7 cells. It also inhibited NF-κB DNA binding induced by LPS in RAW 264.7 cells and prevented MAPK activation caused by LPS by inhibiting the p44/42 MAPKs in the LPS-activated RAW 264.7 cells. The outcome of this study showed that HMP inhibited NF-κB activation and phosphorylation of p44/42 MAPK, hence reducing the LPS-activated production of IL-1, NO, and TNF-α and expression of iNOS and COX-2 genes**.** By modifying the dysregulation of the NF-B pathway, 1′S-1′-acetoxyeugenol acetate (AEA) isolated from *A. conchigera* rhizomes has the capacity to improve apoptosis in MCF-7 cells, as depicted by the decreased expression of various NF-κB-regulated gene targets. Apoptotic resistance resulting from NF-κB activation was eliminated because AEA reduced the phosphorylation of the inhibitor of the κB-kinase complex (Aun et al., 2011).

Several studies have been conducted to evaluate the effect of the bioactive secondary metabolites of *Zingiber* and *Alpinia* species on cellular and humoral immune responses. However, more in-depth studies are necessary to elaborate the mechanism of the bioactive secondary metabolites on different stages of the immune response. Furthermore, bioavailability, preclinical pharmacokinetics, and toxicity studies are necessary before clinical trials can be pursued for development into immunomodulatory agents.

## 8 Conclusion and future directions

Many plants of the genera *Zinger* and *Alpinia* and their secondary metabolites have been investigated for their immunomodulatory effects, especially on the signaling pathways of immune cells. Most studies demonstrated the immunomodulatory activity of the extracts and their constituents through several pathways, such as MAPKs, PI3K/Akt, NF-κB, and Wnt signaling pathways. Subsequently, they modulated cytokines release, such as IL-6, TNF-α, and IL-1β. Among the bioactive metabolites, 6-, 8-, and 10-gingerols, 6-shogaol, and zerumbone from *Zingiber* species and cardamomin, ACA, yakuchinone, rutin, 1,8-cineole, and lectin from *Alpinia* species have demonstrated strong immunomodulating effects and were found to be responsible for the plant potent immunomodulatory properties. Of all the bioactive metabolites, gingerols, shogaols, zerumbone, and cardamomin are specifically present in *Zingiber* and *Alpinia* species and have been extensively studied for their mechanisms to regulate the immune system. However, more mechanistic studies on the regulatory effects of the bioactive secondary metabolites on the immune systems are required for an in-depth understanding of the underlying effects. More studies on the effects of the secondary metabolites in modulating the signaling pathways using experimental cell and *vivo* animal models of immune-related disorders are required together with elaborate preclinical pharmacokinetics, pharmacodynamics, bioavailability, and toxicity studies. Some of these secondary metabolites, particularly gingerols and zerumbone, have the potential for development into immunomodulating agents.
